# Endothelial Dysfunctions in Blood–Brain Barrier Breakdown in Alzheimer's Disease: From Mechanisms to Potential Therapies

**DOI:** 10.1111/cns.70079

**Published:** 2024-11-15

**Authors:** Qian Yue, Xinyue Leng, Ningqing Xie, Zaijun Zhang, Deguang Yang, Maggie Pui Man Hoi

**Affiliations:** ^1^ State Key Laboratory of Quality Research in Chinese Medicine, Institute of Chinese Medical Sciences University of Macau Macao SAR China; ^2^ Department of Pharmaceutical Sciences, Faculty of Health Sciences University of Macau Macao SAR China; ^3^ Department of Cardiology The First Affiliated Hospital of Jinan University Guangzhou Guangdong China; ^4^ The Fifth Affiliated Hospital of Jinan University (Heyuan Shenhe People's Hospital) Heyuan Guangdong China; ^5^ State Key Laboratory of Bioactive Molecules and Druggability Assessment, and Guangzhou Key Laboratory of Innovative Chemical Drug Research in Cardio‐Cerebrovascular Diseases, and Institute of New Drug Research Jinan University Guangzhou China; ^6^ Guangdong‐Hong Kong‐Macau Joint Laboratory for Pharmacodynamic Constituents of TCM and New Drugs Research, and Guangdong Province Key Laboratory of Pharmacodynamic Constituents of TCM and New Drugs Research Jinan University College of Pharmacy Guangzhou China; ^7^ International Cooperative Laboratory of Traditional Chinese Medicine Modernization and Innovative Drug Development of Chinese Ministry of Education (MOE) Jinan University College of Pharmacy Guangzhou China

**Keywords:** Alzheimer's disease, BBB protection, blood–brain barrier, influx and efflux transporter, paracellular permeability, vascular inflammation

## Abstract

Recent research has shown the presence of blood–brain barrier (BBB) breakdown in Alzheimer's disease (AD). BBB is a dynamic interface consisting of a continuous monolayer of brain endothelial cells (BECs) enveloped by pericytes and astrocytes. The restricted permeability of BBB strictly controls the exchange of substances between blood and brain parenchyma, which is crucial for brain homeostasis by excluding blood‐derived detrimental factors and pumping out brain‐derived toxic molecules. BBB breakdown in AD is featured as a series of BEC pathologies such as increased paracellular permeability, abnormal levels and functions of transporters, and inflammatory or oxidative profile, which may disturb the substance transportation across BBB, thereafter induce CNS disorders such as hypometabolism, Aβ accumulation, and neuroinflammation, eventually aggravate cognitive decline. Therefore, it seems important to protect BEC properties for BBB maintenance and neuroprotection. In this review, we thoroughly summarized the pathological alterations of BEC properties reported in AD patients and numerous AD models, including paracellular permeability, influx and efflux transporters, and inflammatory and oxidative profiles, and probably associated underlying mechanisms. Then we reviewed current therapeutic agents that are effective in ameliorating a series of BEC pathologies, and ultimately protecting BBB integrity and cognitive functions. Regarding the current drug development for AD proceeds extremely hard, this review aims to discuss the therapeutic potentials of targeting BEC pathologies and BBB maintenance for AD treatment, therefore expecting to shed a light on the future AD drug development by targeting BEC pathologies and BBB protection.

## Graphical abstract

Blood–brain barrier (BBB) works as a crucial structure separating central nervous system (CNS) and circulating system. The highly organized endothelial junctions and transporters strictly restricts substance exchange across BBB to maintain the homeostasis of CNS. In Alzheimer's disease, BBB breakdown has been widely detected, featured as a series of brain endothelial cell (BEC) pathologies, including increased permeability, abnormal levels and functions of influx and efflux transporters, as well as inflammatory and oxidative profile. The goal for BEC‐targeting treatment in AD is to maintain the integrity of BBB and accelerate the clearance of brain‐derived Aβ species through BBB. BEC‐targeting treatment for AD mainly focuses on regulating a series of pathological processes including inflammation, oxidative stress, autophagy, apoptosis, and several signaling such as angiogenic and Wnt/β‐catenin pathway, as well as modulating Aβ clearance pathway and so on. Targeting various BEC pathologies have shown therapeutic potentials in protecting BBB and improving cognitive functions in AD.

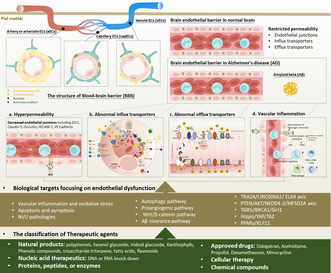

## 1 Introduction

Alzheimer's disease (AD) is the most common form of dementia prevalent among people aged 65 and older. Current drug development mostly focuses on the neuron‐, or microglia‐related pathologies. Particularly, recent studies have highlighted the association between blood–brain barrier (BBB) disruption and prodromal dementia in AD. BBB is a cellular barrier essential for separating brain parenchyma from circulatory system and is particularly important for maintaining brain homeostasis. BBB breakdown has been observed in human throughout the whole progression of AD. MRI has also detected BBB breakdown in preclinical AD or mild cognitive impairment (MCI), along with altered transporters (GLUT1, ABCB1 [P‐gp]). CSF biomarkers for BBB breakdown are also detected in early AD and AD progression when Aβ plaques and NFT are formed [214].

BBB breakdown, accompanied with invasion of neurotoxic, proinflammatory mediators, and impaired molecular transport is demonstrated to impair cognitive functions [12]. Increased permeability of water and small molecules detected by MRI [49], is found in MCI patients and AD, which is considered as biomarker to predict cognitive function [116]. Invasion of peripheral inflammatory mediators and disturbed influx and efflux transporters may accelerate AD progression by aggravating neuroinflammation and Aβ burden. Therefore, apart from the traditional targets for AD, BBB breakdown is probably a potential target for maintaining brain homeostasis and alleviate AD pathologies. Recently, many evidences have indicated the association between BBB impairment and neurodegenerative diseases, especially the therapeutic potential of targeting ECs [274]. Brain endothelial cell (BEC) is the most important part of BBB. Endothelial junctions and transporter proteins respectively consist of paracellular and biochemical barrier of BBB and guarantee the restricted permeability. Decreased level of junctional proteins and transporter proteins in endothelial cells directly lead to BBB leakiness and abnormality of efflux and influx transportation. In addition, pathological BECs exhibit proinflammatory and oxidative profiles, which facilitates the invasion of peripheral immune cells and aggravate neuroinflammation.

We will focus on the contributive role of Aβ pathology in BBB breakdown during AD progression, especially the pathologies of BECs. Previous studies have reported the toxicity of several forms of Aβ including monomer and fibrillary Aβ_1‐42_, soluble monomer of Aβ_1‐40_, and C‐terminal truncated Aβ_1‐24_ fragment on BECs. Fibril and oligomers of Aβ exert direct toxicity on BBB [166]. Soluble monomer Aβ_1‐40_ is pumped out of BBB in paracellular pathway. Therefore, BBB disruption can otherwise promote its clearance. Impaired integrity are also observed in Aβ_25‐35_‐induced BECs [80]. Moreover, the accumulation of zinc in amyloid plaques can induce the hyperpermeability of cerebral microvessels by triggering the accumulation of tissue plasminogen activator (tPA) and plasminogen (PLG) in cerebral microvessels [157].

In this review, we aim to provide a comprehensive overview of the evidence of endothelial alterations in AD from both human tissues and disease models, including the changes in paracellular permeability, BBB influx and efflux transporters, vascular inflammatory and oxidative profile. Meanwhile, we will discuss the possible mechanisms underlying these pathological alterations. In the last, we will summarize the potential therapeutic agents able to ameliorate endothelial pathologies, ultimately enhance BBB integrity and improve cognitive functions in AD, expecting to discuss the therapeutic potentials of targeting BEC pathologies for AD treatment and shed a light on the future AD drug development.

## 2 An Overview of the Physiological Structure of the BBB

In the mature brain microvasculature, the BBB is a tightly regulated selectively permeable cellular barrier that regulate the movement of molecules across the blood and brain parenchyma. The BBB is composed of highly specialized BECs surrounded by pericyte and enwrapped by the endfeet of perivascular astrocytes, together with neurons and microglia and other glial cells in the neuropil, they termed the neurovascular unit. The BBB features are not intrinsic properties of the BECs. During early development, physical contact, and molecular interactions between neuronal and vascular cells (collectively known as neurovascular unit) coordinate the establishment of appropriate BBB features. The exchange of substances between blood and tissue fluid the brain occurs at the capillaries, which are microvessels composed of a continuous monolayer of non‐fenestrated endothelial cells interconnected by junctional proteins surrounded by a basement membrane. This constitutes the basis of the highly selective semipermeable interface of the BBB. Pericytes are embedded in the basement membrane and astrocytes wrap around capillary and pericytes with their endfeet. In adulthood, cells in the neurovascular unit continuously communicate with each other to promote BBB stabilization, maintenance, and immune quiescence. The function of the BBB depends on its properties as a physical barrier, a specific transport system, and a secretory tissue. The physical barrier is achieved by the formation of intercellular tight junction (TJ) and adherens junction (AJ) proteins to seal the capillary lining and restrict paracellular flow, typically only small‐sized polar solutes (MW < 400 kDa, hydrogen bonds < 8) are allowed. At the apical side, transmembrane proteins including occludin and claudins (claudin‐1, ‐3, ‐5 and −12) stabilized by zonula occludens (ZO‐1, ‐2 and ‐3) are the major components of TJ. At the basolateral side, vascular endothelial (VE)‐cadherin (VEC) and platelet endothelial cell adhesion molecule‐1 (PECAM‐1) bind to actin cytoskeleton via catenins (α‐, β‐, γ‐catenin, and p120 catenin) for stabilization. Other components that contribute to endothelial tightness include junctional adhesion molecule (JAM‐A, ‐B, and ‐C), endothelial cell selective adhesion molecule (ESAM) as well as perivascular pericytes and astrocytes. These specialized structures seal the space between adjacent endothelial cells and control the paracellular diffusion of ions and solutes based on their size and charge, while restricting the passage of harmful substances such as pathogens and potentially toxic substances from the blood into the brain.

Essential molecular delivery system across BBB is mediated by vesicular pathways, which contribute to the biomedical barrier in BBB. Receptor‐(Insulin receptor, transferrin receptor, leptin receptor, receptor for advanced glycation end product) ([270]), carrier‐ (GLUT1, GLUT3, CAT1, LAT1, MCT1, CNT2), and adsorptive‐mediated transcytosis (endogenous substances like avidin, cationized albumin, and histones) mediate vesicular transport of larger molecules across BBB [160]. Functional proteins like insulin, transferrin and leptin are mainly delivered into brain by receptor‐mediated transcytosis (RMT). Metabolic substances like carbonhydrates, peptides, proteins, nucleotides, fatty acids, and monocarboxylates are mainly delivered by carrier mediated transcytosis (CMT). Nutrient transporters involved in CMT belongs to solute carrier (SLC) family [95]. Other SLC transporters (OCTs, OATs, OATPs, PEPTs, EAATs, and MAPTs) [214] and ATP‐binding cassette (ABC) family transporters mainly manipulate efflux of drugs or neurotoxic compounds from brain to blood [160].

## 3 Evidence of Pathological Alterations of BECs in BBB Reported in Numerous AD Models: Human, Animal and Cell

### 3.1 BBB Permeability

As mentioned above, TJ and AJ proteins expressed on BECs control the paracellular permeability of BBB. In AD, there is an increase in vascular permeability associated with abnormality of TJ and AJ proteins. In the following section, we will discuss about the changes of BBB permeability and associated alterations of junctional proteins reported in AD patients and animal or cellular models.

#### 3.1.1 Increased BBB Permeability and BBB Leakage

Although current understanding of the mechanisms underlying the development and progression of AD still remain incomplete and uncertain, aging is a well‐established risk factor for AD. Magnetic resonance imaging (MRI) studies using gadolinium (Gd)‐based contrast agents via intravenous injection, which can enhance the visibility of blood vessel and blood flow, is a widely used method for evaluating BBB permeability (*K*_trans_) and could detect subtle BBB damage in vivo. Studies using contrast‐enhanced MRI in living humans have demonstrated that aging‐associated neurodegenerative diseases are characterized by abnormal increase of BBB permeability [5, 55, 142, 151, 225]. Notably, increased BBB permeability occurs with natural aging and is aggravated in patients with mild cognitive impairment (MCI) [55, 142]. These early impairments in BBB barrier function precede observable neuroinflammatory responses, abnormal protein deposition, or neuronal damage, and occur first in the hippocampus with the evidence of pericyte degeneration [142, 151]. In addition, post‐mortem analysis of AD brain tissues showed the significant accumulation of plasma‐derived proteins including immunoglobulins, albumin, fibrinogen, and thrombin in the hippocampus and cortex. It indicates the substantial leakage and breakdown of the BBB in late stages [11, 23, 57, 81, 191, 193, 196, 281]. Invasion of fibrinogen and thrombin into brain can further aggravate neuroinflammation and neurotoxicity [84, 192].

Consistent with human data, in vivo imaging studies in various transgenic AD mouse models also demonstrated paracellular hyperpermeability and BBB leakage at late stage, for example, 8 months in Tg2576 mice and 16 months in 3 × Tg mice [38, 53]. In addition to paracellular hyperpermeability, morphological changes of brain vessel were also observed, including the thickening of cerebral small vessels [199], reduced angiogenesis [108], decreased capillary density [267], reduced cerebral blood flow [41], and increased fibrinogen deposition [35, 61]. These changes are likely associated with increased Aβ deposition within the walls of the microvessels in the brain. Interestingly, the onset of BBB breakdown varies among different types of transgenic AD mouse models, and it appeared to be associated with the specific mutations. Among these mutations, those affecting the presenilin (*PSEN*) appeared to have a more significant impact on BBB integrity, while mutations in amyloid precursor protein (*APP*) and tau (*MAPT*) have comparatively weaker effects. The amount of mutations may also correlate with the onset of BBB damage. 5 × Tg‐AD mice exhibited the earliest onset of BBB damage at 4 months old, while 3 × Tg‐AD mice exhibited BBB damage at 5 months old. Table 1 presents the typical markers used as indicators of BBB leakage, such as increased extravasation of immunoglobulin‐G (IgG), Evans blue, and FITC‐Dextran, in transgenic mouse models of AD ([1]; Aluganti [4, 70]).

**TABLE 1:** The phenotypes of BBB breakdown reported in numerous transgenic AD animal models. (P, Plaques; G, Gliosis; C, Cognitive impairment; the starting timepoints for these AD phenotype characterization are acquired from the website of Alzforum (https://www.alzforum.org/research‐models).)

Genetic background	Mutations	Time point detecting BBB breakdown	Starting time point of AD pathology in Tg‐AD models	BBB breakdown phenotypes	Reference
C57BL/6	5 × Tg‐AD mice APPSwedish (KM670/671NL), APPFlorida (I716V), APPLondon (V717I), PSEN1 (M146L), PSEN1 (L286V)	4 months	P:1–3 (1.5) months G: 1–3 months C: 6 months [170]	BBB breakdown, monolayer composed of isolated ECs revealed increased permeability	[14]
C57BL/6		5 months		BBB leakiness characterized by increased extravasation of immunoglobulin‐G (IgG) and decreased level of Claudin‐5	
C57BL/6	Tg‐SwDl mice APP_Swedish_, APP_Dutch_ (E693Q), APP_Iowa_ (D694N)	9 months	P:3 months G: 6–24 months C: 3, 9, 12 months	BBB leakiness was evaluated by immunostaining of immunoglobulin‐G (IgG) extravasation	[3]
		6 months		Aβ deposition in brain microvessels	
		13 months		Decreased expression of occluding. Isolated EC monolayer reveals increased permeability.	[188]
C57/BL6	3 × Tg‐AD mice APPSwedish, PSEN1 (M146V), and TAU (P301L)	5 months	P:6 months G: 7 months C: 4 months	BBB breakdown characterized as dystrophic astroglial phenotype (decreased levels of GFAP immunoreactive surface and decreased astrocyte process)	[169]
C57BL6/129S		16 months		BBB disruption was only observed at 16 months. Time‐dependent elevation in permeability index and reduction in vascular volume (measured by peak amplitude). Magnetic resonance imaging (MRI) included assessment of hippocampal structural integrity, blood–brain barrier (BBB) permeability (blood–brain barrier perfusion index [BBBi]) and neurospectroscopy	[38]
B6129SF2/J		6 months		Aβ deposition at brain blood vessel was increased at 6 months, accompanied by increased pericyte coverage as well as activated endothelium to facilitate Aβ clearance	[61]
C57BL6/J	APP/PS1 mice APPSwedish, PSEN1 (L166P)	10 months	P: 6–8 weeks (cerebeal amyloidosis), 2 months (neocortex), 3 months (hippocampus) [189] G: 6 weeks, secretion of cytokines and chemokines at later age C: 7–8 months	Decreased expression of ZO‐1 and CD31‐GFAP colocalization. angiogenesis was also inhibited	[108, 267]
C57BL/6					
C57BL/6	APP/PS1 mice APP_Swedish_, PSEN1dE9	6 months	P:4 months (cortex), 6 months (hippocampus) G: 4 months (plaque associated), 8 months (microgliosis) C: 6–10 months	ZO‐1, Claudin‐5 downregulation in cortex and hippocampus. Microvessel density decreases due to Aβ deposition	[267]
C57BL/6	TgCRND8 mice APP_Swedish_, APP_Indiana_	4–12 months	P: 3–5 months G: 3–5 months C: 3 months	Increased permeability BBB breakdown characterized by decreased CBF at 4 months and decreased AQP4 level and upregulation of PDGFRβ at 10 months	[41]
		6 months		Fibrinogen deposition was increasing in brain blood vessel	[35]
C57BL/6	Tg‐ArcSwe mice APP_Arctic_, APP_Swedish_	7 months	P: 5–6 months G: 5–6 months (hippocampus, cortex, thalamus) C: 4–8, 16 months	Increased dextran extravasation	[70]
C57BL/6	APP23 mice APP_Swedish_	12 months	P: 6 months G: 6 months C: 3 months	Obvious thickenings of cerebral small vessel and increased Aβ_40_ deposition in smooth muscle layer	[199]
C57BL6J	PS1_V97L_	6 months	P: 6–9 months (increased Aβ_1‐42_ and the ratio of Aβ_1‐42_/Aβ_1‐40_, and intracellular accumulation Aβ_1‐42_ of oligomer but rare plaques) G: 9 months C: 6–9 months [272]	Increased Evans Blue permeation and decreased TJ proteins	[249]
C57BL/6	Tg2576 mice APP_Swedish_ (isoform 695)	8–12 months	P: 9 months (increased level of Aβ_1‐42_, Aβ_1‐40_), 11–13 months (Aβ deposition in cerebrovascular blood vessels) G: 10–16 months C: 10–15 months [170]	MRI scans showed greater extravasation at 12 months	[53]
C57BL/6J	ApoE‐PON1 double knockout mice	14 months	P: 14 months G: 14 months C: no data	Increased permeation of Evans blue dye into brain	[4]
C57BL/6	PDAPPJ20 mice APP_Swedish_, APP_Indiana_	12 months	P: 1 months (Aβ punctra in hippocampus), 5–7 months (diffuse amyloid plaque), 8–10 months (widespread plaques) G: 6–9 months C: 4 months (learning deficits), 6‐9 months (memory deficits) [170]	Augmented vascular permeability in the hippocampus	[172]
Hybrid C3H/He‐C57BL/6	Tg (APPSwLon) mice APP_Swedish_, APP_London_	No data	Aβ1‐42 is increased at 3–4 months, no deposit at 2 years [103]	NO data	

#### 3.1.2 Alterations of Junctional Proteins

The highly selective and semipermeable diffusion barrier properties of the BBB is primarily determined by the presence of tight junctions (TJs) and adherens junctions (AJs), which are composed of transmembrane proteins expressed on the apical and basolateral side of the endothelial plasma membrane respectively. TJ and AJ proteins are linked to the cytoskeleton machineries that control intracellular signaling networks. The increased BBB permeability observed in AD are likely the result of decreased levels and disorganization of TJ proteins, leading to the formation of gaps, and leaks in the BBB. It is established that claudin‐5 and occludin are essential components of TJs, and any dysregulation of these proteins significantly impact BBB integrity and barrier function [156, 280]. While there have been some studies investigating the changes in TJs and AJs in aging‐related neurodegenerative diseases, the research in human tissues is still relatively limited. In a recent study of post‐mortem analysis, it was observed the protein levels of claudin‐5 and occludin were selectively decreased in the cortical areas but not in the subcortical areas in sporadic AD patients when compared to age‐matched individuals that were normal or cognitively normal with extensive cortical Aβ deposits [246]. These findings were in line with the results from imaging studies in living human, which showed BBB hyperpermeability in MCI/AD patients was region specific, initiated and progressed from cortical areas such as the hippocampus [146]. Notably, cerebrovascular amyloid angiopathy (CAA) and vascular pathology did not appear to be major contributors to the decrease in TJ proteins, although the loss of TJ proteins was associated with the accumulation of insoluble Aβ species, particularly Aβ_40_ (but not insoluble full‐length Aβ_1‐42_), while the soluble Aβ species had not been analyzed in this study. The loss of synaptic marker synaptophysin, which indicated synaptic degeneration, was also found correlating with TJ loss. However, there was no inter‐dependence between these factors, suggesting TJs disruptions may contribute to AD pathogenesis in synergistic and additive manners to Aβ and neuronal pathologies. In addition to TJs, AJs also play crucial roles in stabilizing intercellular junctions and regulating permeability in response to immune signals. Results from another human post‐mortem study indicated the downregulation of AJ protein vascular endothelial (VE)‐cadherin in the brain vessels in both MCI and AD patients, along with increased levels of soluble VE‐cadherin in the blood plasma [44].

In line with human data, decreased expression level of TJ proteins including ZO‐1, Claudin‐5 and Occludin are also observed in numerous transgenic mice, including 5 × FAD mice (4–5 months) [14], APP/PS1 mice (6 months) [108, 267], and Tg‐SwDI mice (13 months) [188]. Apart from decreased level of TJs, downregulation of VE‐Cadherin in brain vessels is observed in AD patients. Similarly, reduced protein level of AJs (VE‐Cadherin, PECAM‐1) were also observed in APP/PS1 mice. Accordingly, increased plasma soluble VE‐Cadherin was observed in AD and MCI patients, as well as in APP‐Tg mice (21 months), which may also indicate disassembly of VE‐Cadherin and loss of VE‐cadherin in brain vessels [105].

Permeability changes are also observed in cellular level. Increased permeability is observed in in vitro isolated EC monolayer [14]. BMECs derived from iPSCs (extracted from AD patients bearing PSEN1 mutation) also show reduced TEER value, and elevated permeability of fluorescein. In parallel, expression of TJ proteins (Claudin‐5, Occludin) was also downregulated [179]. However, another research observes the upregulation of claudin‐5 and claudin‐3 in iPSCs‐derived BMEC from AD patients (PSEN1), although deceased TEER value and decreased level of VE‐cadherin both reflect the decreased endothelial integrity, while no significant difference in the expression of ZO‐1 and occludin. It is still not clear the underlying mechanism and physiological significance behind contradictory alterations of claudins [158]. Upregulation of TJ proteins may serve as the compensatory event upon BBB breakdown. In addition, downregulation of TJ proteins may also helpfully facilitate Aβ clearance though increase of paracellular permeability [92]. Increased permeability is also observed in various brain endothelial cell models under the stimulation of Aβ species. Expression level of ZO‐1 and Occludin are decreased in Aβ_1‐42_‐induced human cell line hCMEC/D3, accompanied by increased permeability of FITC‐dextran across endothelial monolayer [159]. Reduced level of VE‐cadherin is observed in Aβ‐stimulated human umbilical vascular endothelial cell line (HUVEC), as well as phosphorylation of β‐catenin, which is correlated with decreased level of TJ proteins and VE‐cadherin due to the internalization of β‐catenin and dissociation of AJ complex [204]. In Primary rat brain microvascular endothelial cells (rBMVECs), Aβ_25‐35_ also exerted endothelial disruptive effects [26]. Negative finding is also reported. Based on the BBB model established by brain microvessel endothelial cells (BMECs) derived from iPSCs (extracted from normal people with no disease), Aβ_1‐42_ in oligomeric or monomeric forms at nearly physiological concentration (0.5–2 μM) neither showed disruptive effect on BMECs as evaluated by the alteration of TEER, and permeability, even the treatment time was extended into 96 h [171].

**FIGURE 1:**
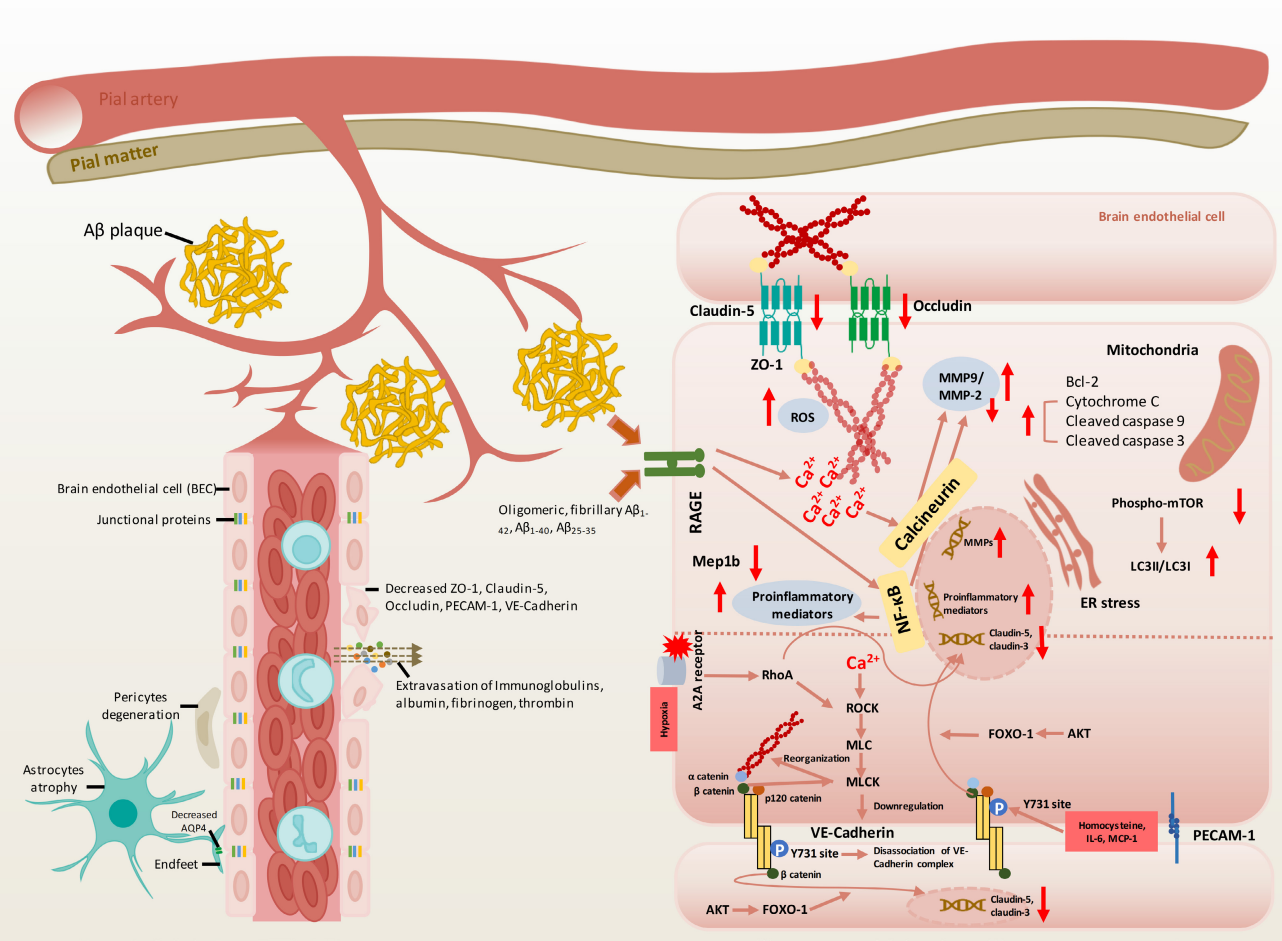
The alterations of paracellular permeability in Alzheimer's disease (AD) patients and numerous AD models. In AD patients and animal models, blood–brain barrier (BBB) becomes leaky, manifested as downregulation of junctional proteins and uncoupling of neurovascular unit cell members. Increased invasion of blood‐derived detrimental mediators like fibrinogen and thrombin further aggravate brain pathologies. In in vitro cell models, numerous amyloid β (Aβ) species induce downregulation of junctional proteins. Activation of NF‐κB pathway and Ca^2+^/Calcineurin‐mediated MMPs upregulation are reported contributing to the downregulation of tight junction proteins, in which mitochondrial apoptosis and increased autophagy are also involved. The reorganization of cytoskeleton and adherens junction, like VE‐Cadherin are observed under the stimulation of hypoxia or inflammatory mediators, in which activation of RhoA/ROCK/MLC/MLCK is involved.

Taken together, as the findings from AD patients and numerous AD models indicate, paracellular permeability of BBB is increased associated with AD pathologies, especially Aβ pathology (Figure 1). Hyperpermeability is accompanied by the downregulation or internalization of TJ and AJ proteins. However, apart from the level of junctional proteins, paracellular permeability of BBB also greatly depends on the structural support from astrocytes and pericytes. In transgenic AD mice, uncoupling of astrocyte endfeet‐endothelial cell or pericyte‐endothelial cell are observed. Decreased GFAP+ immunoreactive surface and astrocyte process [169], decreased colocalization of CD31 and GFAP [108], as well as decreased AQP4 level [41] are observed in 3 × Tg‐AD mice (6–7 months), APP/PS1 (6–9 months) mice, and TgCRND8 (14 months) mice, which indicate structural detachment between astrocyte endfeet and endothelial cells. In TgCRND8 mice, pericytes show abnormal hypertrophic processes with increased immunoreactivity of PDGFRβ [41]. In 3 × Tg‐AD mice (6 months), the coverage of pericyte coverage is also increased, which is explained as a compensatory mechanism to facilitate Aβ clearance through pericyte [61]. Therefore, the paracellular hyperpermeability of BBB under AD conditions is the jointly consequence of degeneration of BECs and dissociation of cell members in neurovascular unit.

### 3.2 Influx Transporters

The normal function of brain greatly relies on the nutrient supply from peripheral blood system through CMT pathway. For instances, glucose transporters (GLUTs), monocarboxylate transporter 1 (MCT1) are required for carbohydrates influx. L‐type amino acid transporter 1 (LAT1), and cationic amino acid transporter 1 (CAT1) are required for influx of peptides and proteins. Concentrative nucleoside transporter 2 (CNT2) is required for nucleotides influx. Major facilitator superfamily domain‐containing protein 2 (Mfsd2a), long‐chain fatty acid transport protein‐1 (FATP1), ‐4, ‐5a, fatty acid‐binding proteins (FABP), and CD36 are required for influx of fatty acids especially docosahexaenoic acid (DHA) [130, 183]. LDLR could also uptake DHA with the assistance of APOE. Brain uptake of low density lipoprotein (LDL) and high density lipoprotein (HDL) is mediated by scavenger receptor class B type I (SR‐BI) which belongs to RMT pathway [183]. Meanwhile, influx transport of functional molecules like insulin and insulin‐like growth factor (IGF) also depend on RMT pathway, which is essential for supporting the brain functions through insulin signaling pathway (mediated by insulin receptor or IGF receptor) [278].

#### 3.2.1 Influx of Glucose

FDG‐positron emission tomography (PET) ([18F]Fluoro‐2‐deoxy‐d‐glucose) have detected impaired regional brain uptake of glucose in MCI and early AD patients [144]. In addition, BMEC‐derived from iPSC extracted from AD patients with PSEN1 mutation also display a reduction in glucose uptake. Impaired uptake of glucose may result from decreased expression of GLUT‐1 and GLUT‐3, which is observed in the post‐mortem of AD patients, although GLUT‐2 is increased and GLUT‐4 is unaltered [215]. More specifically, it is found that GLUT‐1 expresses at a lower level both on BMEC and in hippocampus in AD patients [212]. However, BMEC‐derived from iPSC extracted from AD patients with PSEN1 or PSEN2 mutation showed no significant alterations in glucose transporter isoforms including GLUT1, GLUT3, and GLUT4. In addition, mRNA level of GLUT‐1 is also not altered in BMEC‐derived from iPSC with PSEN1 mutation [158]. Decreased expression of GLUT‐1 has been observed in several types of transgenic AD mice including APP/PS1 mice [175] and mice with APP_Swedish_ (isoform 695) [199]. Findings based on cell models further indicated the association between GLUT downregulation and Aβ pathologies. Decreased expression of GLUT1 in hCMEC/D3 induced by Aβ_1‐40_ and Aβ_1‐42_ [216] and in Aβ_25‐35_‐induced HBMECs, in which reduction of GLUT3 is also observed [239].

In addition to reduced glucose uptake caused by downregulated level of GLUTs, PET‐FDG also detected decreased glucose metabolism in temporo‐parietal regions and posterior cingulate in AD patients, which may have association with Aβ burden indicated by probabilistic maps assessment [63]. However, in other regions like frontal lobes, striatum, and the thalamus, there seems no correlation between alteration of glucose metabolism and Aβ load. In addition, In BMEC with PSEN1 and PSEN2 mutations extracted from familial AD patients, a series of pathological cellular events were observed, including impaired glucose uptake, metabolism (glycolysis), mitochondrial dysfunction, lysosomal acidification, and autophagy [180]. Meanwhile, the findings indicated the stronger relationship between PSEN1 mutations with these pathological events.

#### 3.2.2 Influx of Insulin and Insulin‐Like Growth Factor

Brain insulin is totally dependent on the supply of pancreas [76], and insulin receptor (INSR) on BBB is responsible for the influx of insulin. Other influx transport pathway is also discovered in the absence of insulin receptors [184]. INSR is closely associated with physiological events in various brain cells. For instances, INSR is reported correlated with the expression and activity of various transporters, production and degradation of Aβ, as well as TJ expression in BECs [150]. In hCMEC/D3 cells, knockdown of INSR lead to decreased level of ABCB1, ABCG2, OATP2A1, as well as the efflux activity of ABCB1 [150]. For astrocytes and neurons, insulin modulates the GLUTs expression and localization, which may affect glucose uptake and metabolism. In AD patients, it is observed the decreased uptake of insulin into brain and decreased CSF level of insulin [93], which is probably related to mis‐localized (not on the membrane surface), or reduced INSR on BBB, or decreased receptor affinity for insulin [210]. In severe AD cases, the reduction of INSR at mRNA level estimated 80% is reported [59]. Such decrease in expression of INSR may result from long‐term peripheral hyperinsulinemia occurred in AD adults due to vulnerability of insulin receptor upon diet, plasma glucose level, diabetes, or obesity [8]. While, in Aβ_1‐42_ (oligomeric or monomeric)‐induced hCMEC/D3, increased insulin influx is observed [171]. Decreased INSR and insulin level in AD brain may affect brain metabolism, especially mitochondrial homeostasis with INSR‐mediated signaling including PI3K/Akt and Ras/ERK/MAPK suppressed [140]. In 3 × Tg‐AD mice [64] and APP/PS1 mice [277], decreased level of cerebral insulin receptor‐β is also observed, accompanied with suppression of insulin signaling pathway (PI3K/Akt/GSK‐3β) [277], indicating the hypofunction of insulin pathway in AD cases. IGF‐1 is produced by liver and is transported into brain through IGF‐1 receptor expressed on BECs. Brain IGF‐1 exhibits a widely neuroprotective effect including promoting neurogenesis, stimulating microvascularization and glucose utilization. Overall expression of IGF‐1R was also reduced in AD patients [185], as well as its function. Impaired neuronal IR/IGF‐1R‐mediated signaling pathway was reported featured as decreased IRS‐1/‐2 proteins and decreased phosphorylation of IRS1 in AD patients [141]. Notably, administration of insulin and IGF‐1 could improve metabolic integrity in AD patients [42, 56], as well as improve cognitive function by alleviating synaptic loss, inhibiting tau phosphorylation and brain atrophy in AD patients [10].

#### 3.2.3 Other Influx Transporters

Organic cationic transporters (OCTs) on are responsible for influx of endogenous compounds such as choline, thiamine, and L‐carnitine, as well as influx and efflux of cationic drugs. OCT2 and OCT3 also expresses on neurons and glial cells, mediating the transport of neurotransmitters, which is important for neuronal activity [99]. The reduction of OCT1, OCT2, and OCT3 have been reported in 3 × Tg‐AD mice [195]. The sufficient expression of LAT1 is important for influx of amino acids, as well as some drugs like L‐DOPA and gabapentin [71]. The downregulation of LAT‐1 downregulation was reported in APP/PS1 mice. However, contrary finding is also reported in APP/PS1 mice that the mRNA or protein level LAT1 and its function are unaltered [71]. Fatty acids are essential for the structural development and function of brain. Deficiency of n‐3 fatty acids has been reported correlated with a series of brain pathologies, such as neuroinflammation and cognitive decline [130]. In AD patients, low levels of n‐3 fatty acids in plasma, as well as decreased DHA in several regions including frontal gray, frontal white, and hippocampus have been detected [40, 201]. Meanwhile, CSF levels of fatty acids, cholesterol, and phospholipids are also reduced in AD postmortem brain [147]. Brain fatty acids is transported through various fatty acids transporters. Mice with human *APOE4* mutations show reduced DHA influx [221]. Meanwhile, reduced levels of fatty acids transporters are also detected in AD mice with APP_Swedish_ mutation, in which decreased level of Mfsd2a is found [199]. FATP1 (abluminal brain microvessel) also show reduction (96%) in Aβ_25‐35_‐induced hCMEC/D3 [265]. Therefore, lipid defects in AD conditions associate with reduced influx transport of fatty acids partially due to decreased level of specific transporters. In recent years, the abnormal metabolism of lipid is demonstrated as an important player during the progression of neurodegenerative diseases. Fatty acids have been reported to modulate BBB functions, such as GLUT1 expression and glucose uptake [15]. Notably, a great number of studies have pointed out the therapeutic potentials of maintaining the functions of BBB and neurons by improving brain levels of fatty acids, such as Mfsd2a [22, 152, 235].

#### 3.2.4 Influx of Aβ

Peripheral Aβ can be transported into brain through receptor for advanced glycation end products (RAGE). Upregulation of RAGE is reported in Tg‐SwDl mice [188], APP/PS1 [277], APP_Swedish_‐Tg mice [199], and PS1V97L‐Tg mice [249], which may further aggravate Aβ burden in brain. Increased level of RAGE are also observed in Aβ_1‐42_‐ [118, 206] or Aβ_25‐35_‐ [239] stimulated cell models, accompanied with downregulation of efflux transporters like LRP and ABCB1 (P‐gp), which may severely hampers Aβ clearance through BBB. Increased RAGE induced by Aβ_1‐42_ is also demonstrated as the causative role in upregulation of MMPs and downregulation of junctional proteins [226]. In addition, organic anion‐transporting polypeptide 1a4 (OATP1A4) was reported participating in Aβ uptake, inhibition of which alleviates Aβ burden in brain [51, 87].

**FIGURE 2:**
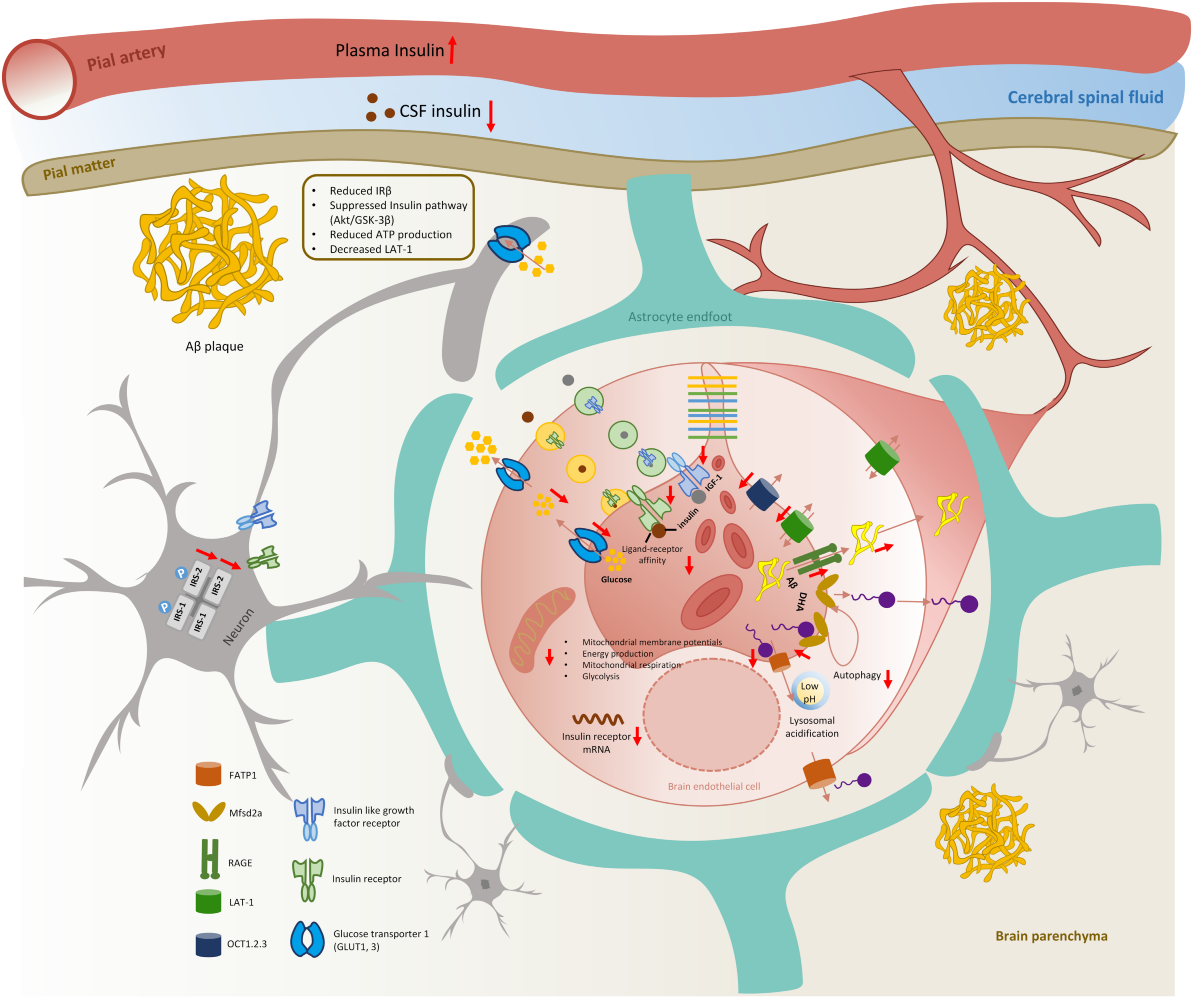
The alterations of influx transporters in Alzheimer's disease (AD) patients and numerous AD models. Influx transporters are responsible for supplying the nutrients and energy substances like glucose, which are necessary for the activity of brain cells. In AD patients and AD animal models, hypofunction, and decreased level of glucose transporter 1, 3 (GLUT1, 3), as well as insulin receptor or insulin like growth factor receptor (IGFR) are commonly observed, accompanied with decreased glucose uptake, energy metabolism, and reduced insulin signaling pathway. Similarly, cerebral spinal fluid (CSF) levels of insulin is decreased, while plasma level is increased. Unsaturated fatty acids influx by Mfsd2a and FATP1 are also showed a decrease, which indicates the loss of protection on blood–brain barrier (BBB). However, in the contrary, the expression level and activity of receptor for advanced glycation end products (RAGE) are increased, which would result in increased Aβ influx that aggravate brain Aβ pathology.

Taken together, as shown in Figure 2, various influx transporters responsible for influx of nutrients are downregulated in AD patients and other AD models, which may result in decreased uptake of glucose, insulin, as well as fatty acids. Accordingly, low level of glucose, insulin, and fatty acids subsequently lead to hypometabolic state and abnormality of cellular processes in AD brain. Moreover, these pathological conditions mutually induce each other, thus accelerating the disease progression. For instance, insulin and fatty acids could affect glucose metabolism by modulating mitochondrial respiration and GLUTs expression and localization. Therefore, the alterations of these influx transporters may be a detrimental factor for neuronal degeneration in AD brain. In addition, cell models also indicated the causative relationship between Aβ pathology or PSEN mutations and the reduction of these influx transporters and related abnormalities of physiological processes.

### 3.3 Efflux Transporters (Correlated With Aβ Efflux)

#### 3.3.1 LDL Receptors/PICALM/ATP‐Binding Cassette (ABC) Transporters

In individuals with early AD, decreased function of efflux transporter ABCB1 (P‐gp) is detected by decreased intensity of verapamil (P‐gp ligand)‐PET in brain [144]. The reduction of several BBB efflux transporters, such as ABCB1 (P‐gp), ABCG2, OATP2B1, and ENT1 in AD patients is detected in Aβ‐accumulated brain districts like gray matter and hippocampus, while much abundant in cerebellum that are not affected by AD pathology [212]. In BMEC derived from iPSC extracted from AD patients with PSEN1 mutation, the protein and mRNA level of ABCB1 (P‐gp) were decreased [158]. Moreover the efflux activity of ABCB1 (P‐gp) is also reduced [179, 180]. Therefore, it indicates that the alterations of efflux transporter may correlate with Aβ pathology. In Tg‐SwDl mice, reduced level of efflux transporters including ABCB1 (P‐gp), ABCG2, and ABCC1 were also observed [188]. Decreased level of LRP‐1 is widely found in 3 × Tg‐AD mice [64], mice with APP_Swedish_ mutation [199], and APP/PS1 mice [248]. Brain‐derived Aβ undergoes abluminal LDL receptor‐related proteins 1 (LRP1) reuptake and phosphatidylinositol‐binding clathrin assembly protein (PICALM)‐mediated transcytosis and is eventually removed out through several efflux transporters of ABC family [175] (ABCB1 [P‐gp], ABCG2, ABCG4, ABCC1) located at luminal side [214]. Therefore, it may indicate the decreased Aβ clearance through LRP1 under AD conditions. In vitro models also found the alteration of efflux transporters under Aβ stimulation. For example, in isolated rat brain capillary, Aβ_1‐40_ downregulates ABCB1 (P‐gp) expression and transport activity [73]. In hCMEC/D3, Aβ_1‐42_ induces ABCB1 (P‐gp) downregulation at mRNA and protein level, even its transcriptional promoter is inhibited [13]. However, Aβ may not be the causative factor in AD‐induced alteration of efflux transporters. In APP/PS1 mice, LRP1 decrease is observed in both cortex and hippocampi starting at 4 months when Aβ plaques are formed in cortex, but not in hippocampus [248]. Reduction of ABCB1 (P‐gp) function (50%) is detected before the occurrence of Aβ deposition in APP/PS1 mice [283].

#### 3.3.2 Other ABC Transporters

ABCA1 [253], ABCG1 [232], ABCG4 [52], ABCA7 [102], ABCC1 [175], as well as ABCG2 [244] are mainly responsible for cholesterol efflux and also involved in BBB‐mediated Aβ clearance [19, 167]. Impaired cholesterol efflux capacity through these transporters in AD patients has been reported, accompanied with reduced Aβ clearance [133]. In AD patients, mRNA and protein level of ABCA1 is reported positively correlated with severity of dementia [2], which may be due to ABCA1‐mediated disruption of lipid architecture thus disturb cellular activities. ABCA1 is essential for APOE lipidation and APOE‐dependent Aβ clearance through LRP1, LDLR, and VLDLR [29]. APOE alleles exhibit different lipidation activity, thus have different impact on Aβ clearance velocity. APOE2, APOE3, and APOEJ are more efficient in assisting Aβ clearance compared to APOE4 allele [276]. ABCA1 upregulation in severe AD cases may also act as a compensatory mechanism intensifying Aβ clearance activity. In APOE4 targeted replacement mice, activation of ABCA1 could reduce APOE4‐mediated Aβ accumulation and tau phosphorylation [24]. However, in APP/PS1 mice, ABCA1 was decreased, accompanied by decreased level of APOE, LRP1, LDLR and brain Aβ accumulation and plasma level of cholesterol are increased [264], indicating impaired Aβ clearance activity and cholesterol transport. ABCA7 mediates Aβ clearance through microglia and BMECs [102]. The association between ABCA7 genetic variants and AD risk has been reported in different populations [120, 145, 181]. ABCC1 is responsible for the removal of HNE (4‐hydroxy‐2‐transnonenal, lipid peroxidation products). The production of HNE is related to the production of radical species induced by Aβ and could exert neurotoxicity by impairing the structure and functions of synaptosomal membrane proteins [32]. In AD patients, reduced ability of ABCC1 is reported accompanied with elevated level of HNE in brain [119, 134].

In addition, reduction of other efflux transporters like plasma membrane monoamine transporter (PMAT) and multidrug and toxic compound extrusion proteins (MATE1) are also detected in capillaries from AD patients, which may lead to neurotoxicity by facilitating the accumulation of antipsychotic drugs in brain [195].

**FIGURE 3:**
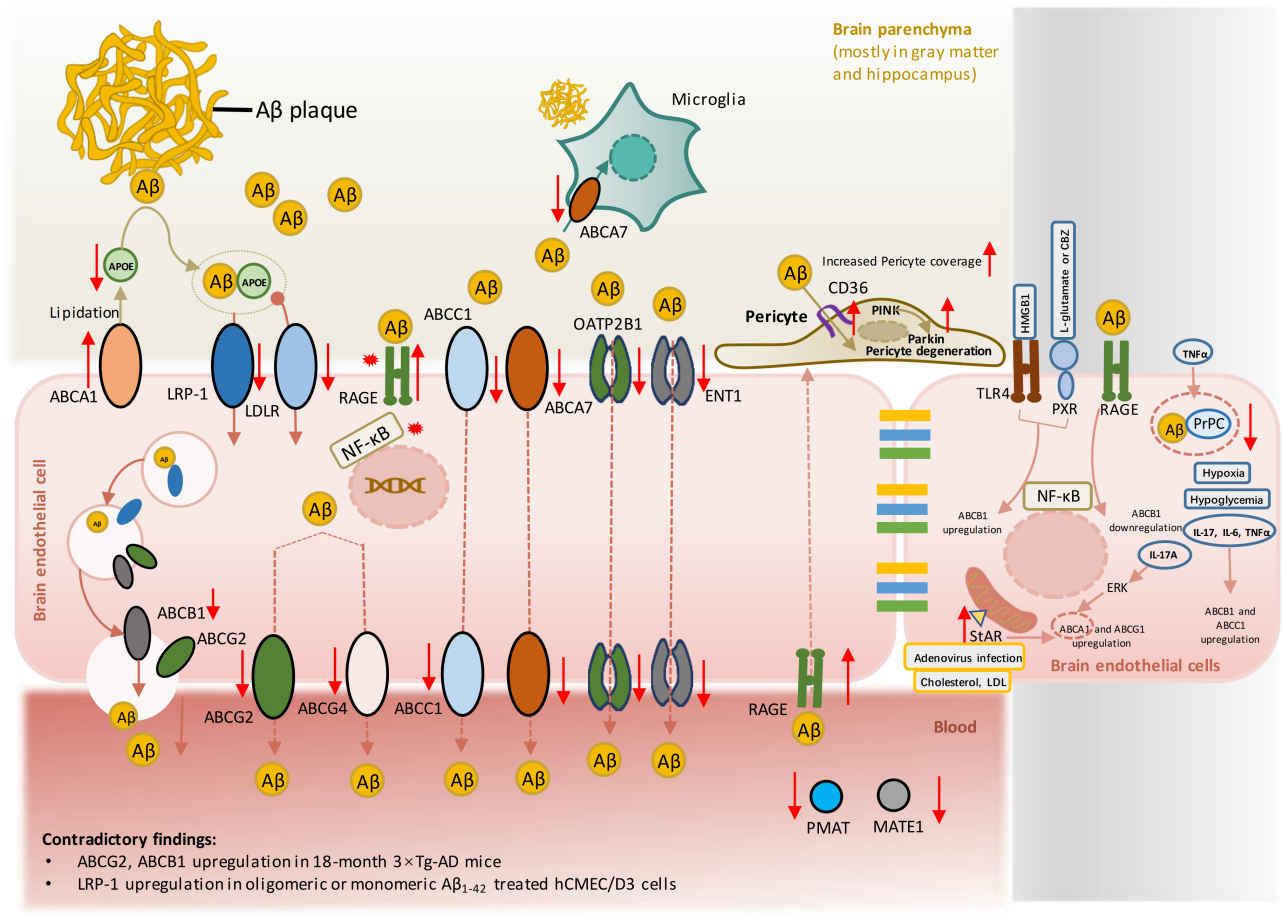
The alterations of efflux transporters in Alzheimer's disease (AD) patients and numerous AD models. Efflux transporters expressed on blood–brain barrier (BBB) locate at both luminal and abluminal sides of brain endothelial cells, mainly responsible for pumping out brain‐derived toxic factors and excluding blood‐derived neurotoxic factors or drugs. Decreased expression and activity of efflux transporters that are responsible for amyloid β (Aβ) clearance are observed in gray matter and hippocampus where Aβ plaques distribute. Microglia ABCA7‐mediated Aβ reuptake is also impaired. However, opposite findings of improved Aβ clearance are also reported, for instance of increased ABCA1 mediated lipidation of APOE and pericyte CD36‐mediated Aβ reuptake. Upregulation of efflux transporters are also observed in in vitro cell models that are induced by inflammatory mediators or Aβ and late stage of AD mouse models.

Taken together, as shown in Figure 3, various efflux transporters responsible for Aβ are downregulated especially in Aβ deposition‐districts in AD patients, and numerous AD models, indicating the association between Aβ pathology and alterations of efflux transporters and a subsequent accumulation of Aβ. However, compensatory increase of Aβ efflux against Aβ overload is also reported. For instances, in AD patients, there is a positive correlation between ABCA1 level and AD severity. In hCMEC/D3, oligomeric or monomeric Aβ_1‐42_ stimulates upregulation of LRP1 [171]. In addition, in AD animal models, compensatory increased Aβ clearance mediated by increased pericyte coverage and activated endothelium [61], as well as upregulation of CD36 in pericytes [110] are also reported. Such defensive increase of Aβ efflux transporter may occur during the whole disease progression. For example, in 3 × Tg‐AD mice (Aβ plaques are formed at 6‐month‐old, preceding which cognitive impairment occurs [3–6 months]), ABCA1 are upregulated during 3–6‐month‐old (before Aβ plaques are formed but cognitive impairment occurs) to maintain the equilibrium of Aβ influx and efflux transport against increased Aβ influx due to downregulated LRP1 and upregulated RAGE. At 18 months, ABCG2 and ABCB1 (P‐gp) are upregulated thus achieving net increase of Aβ efflux [52]. Therefore, although the alteration of various efflux transporters varies in a disease stage‐dependent pattern, while the net Aβ efflux activity might elevate as the disease gets severer.

### 3.4 Vascular Inflammation and Oxidative Stress

Brain vasculature exhibits proinflammatory and oxidative profile during the progression of AD. Increased production of proinflammatory mediators including cytokines, chemoattractant, adhesion molecules, and MMPs would accelerate the impairment of BBB permeability and transport system, thereafter contributing to BBB leakiness. Moreover, the upregulation of chemoattractant and adhesion molecules facilitate the infiltration of immune cells, which aggravating neuroinflammation and neuronal death through proinflammatory secretions. In the following sections, we will discuss about the specific proinflammatory and oxidative profile of brain vasculature reported in AD patients, animal models or cellular models.

#### 3.4.1 Proinflammatory Mediators

Under AD conditions, vascular inflammation and oxidative stress manifested as activation of NFκB pathway interplay with various endothelial pathological events including hyperpermeability, alterations of influx and efflux transporters. AD brain exhibits a highly inflammatory status. Brain level of proinflammatory cytokines such as TNF‐α, IL‐1, IL‐6 and IL‐8, and MMPs (MMP‐3 and MMP‐9) are increased in AD patients [115]. Upon the stimulation of Aβ, isolated brain microvessels from AD patients secrete increased level of pro‐inflammatory factors including thrombin [255], TNFα, nitric oxide (NO) interleukins, and MMPs (MMP‐9) [219]. In vitro stimulation of Aβ_1‐40_ or Aβ_1‐42_ hCMEC/D3 cells upregulates mRNA expression of MCP‐1, GRO, IL‐1β, IL‐6 [224], and increased IL‐1β secretion [159]. Therefore, it may indicate the causative role of Aβ in vascular and endothelial inflammation. In vitro BMEC model (hCMEC/D3) has also demonstrated the important role of semicarbazide‐sensitive amine oxidase (SSAO/VAP‐1) in stimulating endothelial inflammation and angiogenesis that further lead to BBB hyperpermeability and leukocyte adhesion [203]. By evaluating the plasma and cerebrovascular tissues from AD patients, enzymatic activity of SSAO/VAP‐1 is found enhanced. In transgenic AD mouse model, it is also found that overexpression of proinflammatory cytokines or endothelin‐1 (ET‐1) could be mediated by brain endothelial RAGE [47, 54]. Especially, disrupted microvessels near Aβ plaque area exhibit increased RAGE, MMP secretion in 5 × FAD mice [100]. In addition to the brain vasculature, several factors correlated with vascular inflammation and oxidative stress, such as IFN‐γ‐induced protein 10 (IP‐10), pregnancy‐associated plasma protein A (PAPP‐A), total and intact proinsulin, glutathione S‐transferase alpha, and MMP‐1 are increased in the peripheral vascular system during LOAD progress [85].

#### 3.4.2 Chemoattractant, Adhesion Molecules, and Infiltration of Immune Cells

A group of chemoattractant were upregulated in AD patients, including P‐selectin, E‐selectin, ICAM‐1, VCAM‐1, and plasma vesicle associated protein (PLVAP), among which ICAM1 and VCAM1 mediate firm adhesion, P‐selectin, and E‐selectin mediate the rolling of leukocytes along the endothelium, and PLVAP participates in transmigration across ECs [173]. A positive correlation between VCAM‐1 and severity of dementia has been identified by MRI [115]. Increased expression of E‐selectin, P‐selectin, ICAM‐1, and VCAM‐1 is also detected in brain vessels of the cortex, hippocampus, amygdala, meninges, and choroid plexus in transgenic AD mice bearing APP or tau mutations [21]. Neutrophils has been detected in meningeal or cortical blood vessels or even in brain parenchyma in AD patients indicated by increased neutrophils‐specific immunoreactivity of Capthepsin G [202]. In addition, macrophage [82, 263], CD4^+^, and CD8^+^ T cells [128] are also detected in brain parenchyma in AD patients. Therefore, it indicates that the high level of chemoattractant and increased immune cells infiltration in AD brain may participate in the neuroinflammation and disease progression. Aβ pathology may a key factor triggering the infiltration of immune cells. Increased monocyte migration is also observed in Aβ‐induced brain endothelial cell layer extracted from AD patients [198]. Meanwhile, increased transendothelial migration of monocyte (HL‐60, THP‐1) across human brain endothelial cell line model (HBMVEC) in induced by Aβ_1‐40_, such effects are probably mediated by RAGE and PECAM‐1 [58, 67]. In 5 × FAD mice, the infiltration of neutrophils through brain endothelium into brain parenchyma especially in Aβ‐rich cortical regions is also detected by two‐photon laser scanning microscopy [168].

#### 3.4.3 Oxidative Stress

As CellRox (R) and DCFDA assays indicates that, BMEC derived from iPSC from AD patients with PSEN1 mutation showed highly oxidative status, and the effect is similar to the stimulation of 1 mM pyocyanin, a known oxidative stress inducer [179, 180]. ROS overproduction is also observed in in vitro Aβ‐induced hCMEC/D3 cells [28].

**FIGURE 4:**
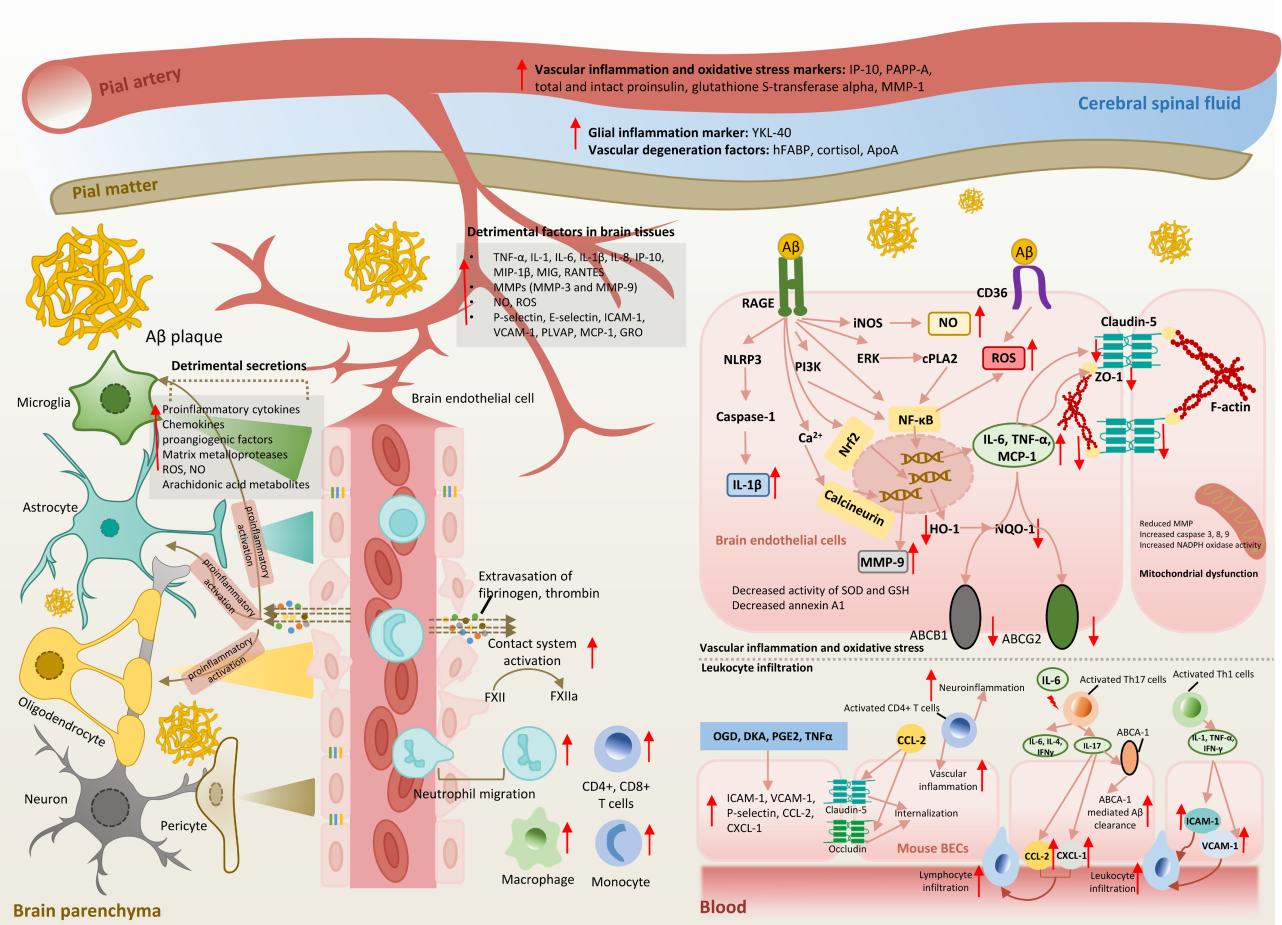
The alterations of vascular inflammation and oxidative stress in Alzheimer's disease (AD) patients and numerous AD models. Vascular inflammation and oxidative stress are detected accompanied with leaky BBB in AD cases. Increased proinflammatory cytokines, chemokines, matrix metalloproteases (MMPs) et al. are observed in brain tissues. Leaky BBB facilitates the entrance of blood‐derived fibrinogen, thrombin and infiltration of immune cells which further trigger the glial inflammation and pericyte activation through cell–cell interaction. Vascular inflammation marker proteins are detected increased in cerebral spinal fluid (CSF) and plasma. Vascular inflammation can be stimulated either by detrimental glial secretions or amyloid β (Aβ). Activation of RAGE by Aβ initiates Ca^2+^‐Calcineurin‐, NF‐κB‐, Nrf2 signaling‐, and decreased anti‐oxidative capacity‐mediated vascular inflammation and oxidative stress. Increased endothelial production of proinflammatory mediators, reactive oxygen species (ROS), nitric oxide (NO), and MMPs further downregulate junctional proteins and Aβ efflux transporters. Infiltration of immune cells across in vitro endothelial monolayer is observed mostly under the stimulation of proinflammatory mediators and hypoxia, possibly resulting from increased production of adhesion molecules and chemokines. Activated T cells, such as CD4^+^ T cells, T helper 1 (Th1) cells, and Th17 cells secrete a series of proinflammatory mediators which also stimulate vascular inflammation and immune cell infiltration.

Taken together, as shown in Figure 4, brain vessels in AD patients or AD animal models exhibit a proinflammatory and oxidative profile, characterized as increased production of proinflammatory cytokines, chemoattractant, adhesion molecules, MMPs, NO, and ROS, which are detrimental to endothelial integrity and transporter system and may lead to BBB leakiness. Inflammatory endothelial cell layer facilitates the infiltration of immune cells into brain. These immune cells further trigger the neuroinflammation and cognitive deficits through specialized proinflammatory secretion profile. In addition, several plasma proteins in AD cases that are related to vascular inflammation and BBB hyperpermeability have also been found, eventually contributing to neuroinflammation and neuronal death. For example, increased perivascular accumulation of blood‐derived fibrinogen, thrombin is observed in AD patients [9] and the colocalization of these blood‐derived factors with Aβ is also reported [214]. It has been demonstrated in AD patients and animal models that leaky BBB facilitates the infiltration of fibrinogen, and fibrin are formed mainly around Aβ deposition district which then trigger the overproduction of ROS in microglia and aggravating neuroinflammation and neuronal death [137]. Activation of plasma protein factor XII (FXII)‐driven contact system is also observed in the plasma of AD patients, Tg6799 mouse, and Aβ_1‐42_‐induced C57BL/6. The causative role of circulating Aβ_1‐42_ in inducing contact system activation is demonstrated, the product of which, bradykinin in peripheral system or brain parenchyma could further induce BBB hyperpermeability, vascular degeneration, cytokine release, and neuroinflammation [262]. In TgCRND8 AD mice, it is found that deletion of FXII could reduce fibrinogen deposition, neuroinflammation, and neurodegeneration [35].

## 4 Probable Signaling Pathways Responsible for Endothelial Pathologies

### 4.1 Paracellular Hyperpermeability

The toxicity of Aβ_1‐42_ oligomers on expressions of junctional proteins and endothelial integrity are widely investigated on immortalized mouse brain microvessel endothelial cell line, bEnd.3 cells. Apart from Aβ_1‐42_ oligomers, fibrillary Aβ_1‐42_, Aβ_1‐40_ monomer and dimer could also induce the reduction of claudin‐5 and occludin in bEnd.3, which may act as a defensive mechanism to increase Aβ transport as mentioned above [92, 118]. Abundant evidences about the molecular mechanisms underlying the alterations of junctional proteins induced by Aβ_1‐42_ are widely reported based on bEnd.3 cells. Among them, vascular inflammation acts as the causative role for other endothelial pathologies. Endothelial inflammation characterized as NFκB activation and ROS overproduction, was triggered by Aβ_1‐42_ oligomer and demonstrated capable of inducing the downregulation of ZO‐1 and Claudin‐5 [206]. Overexpression of proinflammatory cytokines (TNF‐α, IL‐1β, and IFNγ) in bEnd.3 directly downregulates the level of junctional proteins (ZO‐1, Claudin‐5), while ABCG2 and ABCB1 are increased, probably as a defensive mechanism for Aβ clearance [222]. Additional treatment of anti‐inflammatory messenger annexin A1 restores expression of ZO‐1 and Claudin‐5 [162]. Chemoattractant CCL2 increases the internalization of Claudin‐5 and Occludin, thereafter leads to decreased membrane level of tight junctions and TEER reduction [209]. Apart from inflammatory responses, TJ downregulation is also the downstream events of several cellular pathological processes induced by Aβ. Activated autophagy (increased LC3II/LC3I and decreased phosphorylation of mTOR) [30], increased mitochondrial apoptosis (decreased Bcl‐2, increased Bax, cytochrome C, and increased cleavage of caspase‐9 and caspase‐3) and activated death receptor signaling pathway (increased cleavage of caspase‐8 and caspase‐12) [117] are all reported involved in inducing TJ downregulation. Endoplasmic reticulum stress is found involved in Aβ_1‐42_ oligomer‐induced TJ downregulation and treatment of ERS inhibitor, salubrinal could prevent TJ downregulation [34]. MMPs is widely acknowledged as an important role for TJ degradation. Increased secretion of MMPs (MMP‐9, MMP‐2) can be induced by Aβ_1‐42_ triggered activation of Ca^2+^‐calcineurin signaling, leading to TJ downregulation, and endothelial barrier dissociation [100]. Apart from Ca^2+^‐calcineurin pathway, it is reported that activation of NFκB pathway and combination between Sphingosine‐1‐phosphate (S1P) and receptor S1PR [98] can also upregulate MMPs and its associated effects.

The Effect of Aβ on AJ proteins is rarely studied in bEnd.3 cells. Therefore, we'll talk about the evidences in bEnd.3 cells about the signaling related to AJ alterations stimulated by other inducers. In lanthanum chloride‐induced stroke model, intracellular Ca^2+^ elevation in bEnd.3 is involved in the loss of VE‐cadherin and reorganization of cytoskeleton, accompanied by activated ROCK/MLC/MLCK pathway [249]. Increased phosphorylation of VE‐cadherin at Y731 site, dissociation of VE‐cadherin/β catenin complex [16] and further nuclear translocation of β catenin are observed in homocysteine‐induced hyperhomocysteinemia model. Homocysteine‐induced nuclear translocation of β catenin further inhibit the expression of Claudin‐5 and Claudin‐3 [17]. The activation of AKT/FoxO1 is identified as an upstream event for nuclear translocation of β‐catenin and increased accumulation of β‐catenin and FoxO, eventually inhibiting the gene expression of claudin‐5 [139]. In addition, nuclear translocation of β catenin can also be induced by IL‐6‐ and MCP‐1 [240]. Apart from modulating the expression of claudins, β‐catenin also modulates cytoskeleton via non‐muscle myosin light chain kinase (nmMLCK) [97] and vascular integrity via Wnt/β‐catenin signaling [107]. Leukocyte infiltration can also be suppressed [18]. Activation of RhoA signaling pathway is reported involved in the downregulation of junctional proteins. Activation of RhoA signaling mediated by hypoxia‐induced activation of adenosine A2A receptor potently downregulate the expression of claudin‐5 and VE‐cadherin and reorganize cytoskeleton architecture [247]. Connexins (Cx) are a group of proteins forming the gap junction channels for intercellular communication. It is reported that Cx43 is involved in stabilizing TJ proteins (ZO‐1 and claudin‐5) under CoCl_2_‐induced hypoxia condition. Similar stabilizing effect of connexin 43 on BBB is also observed in vivo. Cx43 is also involved in CoCl_2_‐induced HIF1α activation, AKT phosphorylation, and VEGF‐mediated angiogenesis [257]. Overexpression of BACE1 in endothelial cells is also observed involved in the reduction of Occludin in CAA models [36]. Metalloprotease meprin β (Mep1b) is able to cleave cell‐adhesion molecules, dysregulation of which is associated with BBB breakdown. Mep1b dysregulation is reported in AD. In bEnd.3, decreased expression of TJ proteins and monolayer integrity are observed followed by the overexpression of Mep1b [66]. Mep1b knock‐out in animal models in turn increase the TJ expression and BBB integrity (Figure 1).

### 4.2 Alteration of Efflux Transporters

Activation of RAGE/NFκB is responsible for Aβ_1‐42_‐induced ABCB1 (P‐gp) downregulation in cells [164] and on the luminal membrane of the brain capillary endothelium [164]. However, NFκB activation is also involved in the upregulation of ABCB1 (P‐gp) in HMGB1‐ [227] in L‐glutamate‐ or CBZ‐ [91] stimulated cells respectively. HMGB1 and L‐glutamate or CBZ mediate NFκB activation respectively through TLR4/RAGE and Pregnane X receptor (PXR). RAGE is not only involved in ABCB1 (P‐gp) downregulation. Under chronic hypoxic and hypoglycemic conditions, upregulation of RAGE mRNA and downregulation of LRP‐1 mRNA co‐occur [247]. Contrary finding is also reported that upregulation of RAGE is also responsible for Aβ‐induced activation of LRP‐1/PICALM/Rab11/ABCB1 (P‐gp), which further facilitates Aβ efflux [211]. Proinflammatory mediators are main inducers for NFκB activation. Therefore, downregulation of efflux transporters occurs after the stimulation of proinflammatory mediators. For example, LPS could downregulate ABCB1 (P‐gp), ABCG2, and ABCC1 [175]. Cellular prion protein (PrPC) is located on endothelium and involved in LRP‐1‐mediated Aβ efflux. In mouse brain endothelial cell line (MBEC4), TNF‐α treatment downregulates PrPC, accompanied with decreased Aβ clearance as indicated by decreased colocalization of intracellular Aβ and PrPC [254]. However, cytokine complex (IL‐17, IL‐6, TNF‐α) could increase the expression and transport activity of ABCB1 (P‐gp) (Rhodamine‐123) and ABCC1 (BCECF‐AM) [222]. Proinflammatory mediators can also affect the expression of ABCA1. In APP/PS1 mice with IL‐17A overexpressed and in bEnd.3 cells with external treatment of IL‐17A, ABCA1 is upregulated [250], in which ERK activation is involved [250]. Steroidogenic acute regulatory protein (StAR), a mitochondrial cholesterol transporter [153] is reported involved in modulating ABCA1 expression. In adenovirus infection [153] or cholesterol and LDL‐induced bEnd.3 cells [154], increased expression of ABCA1 can be induced by StAR activation (Figure 3).

### 4.3 Vascular Inflammation and Oxidative Stress

Aβ_1‐42_‐induced endothelial inflammation in bEnd.3 cells is manifested as activated iNOS and increased NO production, as well as increased secretion of proinflammatory cytokines (IL‐6, TNF‐α, and MCP‐1) [206]. RAGE/NFκB is the main pathway that mediates Aβ‐induced endothelial inflammation. Activation of NLRP3/cleaved caspase 1/IL‐1β signaling is also involved in Aβ‐induced endothelial inflammation (Aβ_25‐35_) [245]. Aβ_1‐40_ could intensifies IL‐1β‐induced vascular inflammation through activating PI3K and NFκB in vascular smooth muscle cells [223]. Apart from proinflammatory effects, Aβ_1‐42_ also impairs anti‐inflammatory capacity, in which decreased annexin A1 (anti‐inflammatory messenger) is reported [162]. Aβ_1‐42_‐induced MMP upregulation is demonstrated mediated by activation of Ca^2+^‐calcineurin pathway [100].

NFκB nuclear translocation, decreased activation of Nrf‐2/NQO‐1/HO‐1 pathway and mitochondrial dysfunction (reduced MMP and increased level of caspase 3,8,9) [123] are involved in Aβ_25‐35_‐induced oxidative stress including increased NADPH oxidase activity [60], ROS production and inhibited antioxidant enzyme activity (SOD, GSH). In addition, Aβ_1‐42_‐induced ROS overproduction is also accompanied with activation of ERK and cPLA2 [218]. CD36 is also reported involved in Aβ_1‐40_‐induced ROS overproduction [163].

Leucocyte infiltration across bEnd.3 monolayer is rarely reported under AD conditions, but in other disease models, such as OGD [27, 79]‐induced ischemia model, DKA‐ [39], PGE2‐ [165], and TNF‐α [126]‐ induced inflammation models. Upregulation of adhesion molecules like ICAM‐1 [165], VCAM‐1 [104], P‐selectin, and chemoattractant like CCL‐2, and CXCL‐1 [236] are observed in activated bEnd.3 cells. Immune cells that are reported infiltrating across bEnd.3 monolayer mainly include monocyte (THP‐1) (5%) [78], macrophage (IC21) [27], and lymphocyte (80%) (CD4^+^ T cell [88], CD8^+^ T cell [230]) [178]. Among different types of lymphocytes, Th1 and Th17 are most studied. Activated CD4^+^ T cells in CNS release a series of inflammatory cytokines aggravating neuroinflammation and vascular inflammation. T cells can be activated by cytokines. IL‐6 secreted from muramyl dipeptide (MDP)‐induced bEnd.3 further activates Th17 cells and lead to the secretion of a series of cytokines (IL‐17, IL‐6, IFNγ, and IL‐4) [132]. Among them, IL‐17 further upregulates CCL2 and CXCL1 on BMEC membrane, which facilitates lymphocyte infiltration [236]. Activated Th1 cells secrete IL‐1, TNF‐α, and IFN‐γ, which would aggravate leukocyte infiltration by upregulating ICAM‐1 and VCAM‐1 and other adhesion molecules [124] (Figure 4).

## 5 Biological Targets: A Focus on Endothelial Dysfunction

### 5.1 Overview of Intervenable Endothelial Factors

**FIGURE 5:**
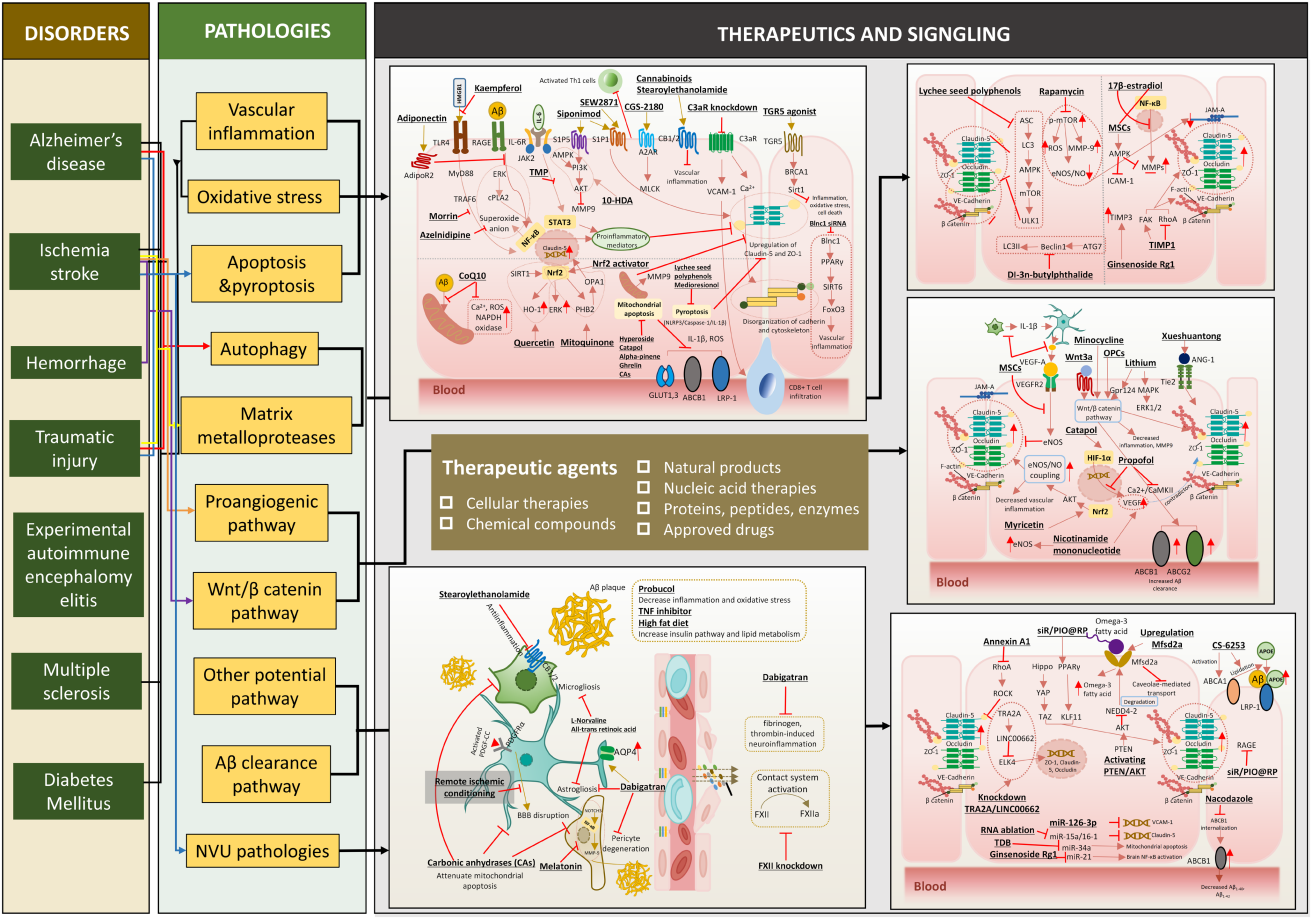
Therapeutic strategies of targeting endothelial pathology in treating Alzheimer's disease (AD). The attempts of therapeutic strategies by protecting blood–brain barrier (BBB) integrity and function in AD in vivo models showed the effective outcomes including reduced Aβ burdens and improved cognitive functions. We also included some BBB protection strategies in other disease models, such as ischemia stroke, traumatic brain injury, and experimental autoimmune encephalomyelitis. BBB protection strategies mainly aim to alleviate neurovascular unit cell members mutual inducement, vascular inflammation, and oxidative stress, prevent cell apoptosis, inhibit autophagy pathway, reduce matrix metalloproteases, modulating proangiogenic pathway, activate Wnt/β‐catenin pathway, modulating mRNA expression, and other pathways. After treatment, upregulated junctional proteins, increased Aβ clearance, improved cell survival, and decreased vascular inflammation and oxidative stress contribute to the recovery of BBB integrity and function.

The manifestations of AD‐induced BBB damage mainly include increased paracellular permeability, abnormality of influx or efflux transporter and vascular inflammation that eventually leads to abnormal substance transport, including Aβ clearance and uptake of essential molecules. Therefore, to maintain the normal paracellular permeability, along with expression and activity of transporters are essential to protect brain homeostasis and reduce Aβ burden. We tabulated the reported effective therapies capable of protecting BBB integrity under numerous disease conditions, such as AD, ischemia stroke or hemorrhage, traumatic brain injury (TBI), and inflammation‐related disease such as experimental autoimmune encephalomyelitis (EAE) and multiple sclerosis (MS) (2019–2023) (Table 2). BBB protective compounds can be grouped into several classifications including natural products (herbal or human body‐derived compounds), peptide or proteins, nucleic acid therapeutics, chemical compounds, or some approved drugs and cellular therapy. In AD models, the strategy for BBB protection mainly aims to upregulate TJ or AJ proteins [245], enhance the expression of Aβ clearance transporters (LRP‐1, ABCB1 [P‐gp]) and ApoE clearance pathway [14], as well as other endothelial pathologies. The graphic illustration is shown in Figure 5.

**TABLE 2:** The list of compounds that have therapeutical potentials on brain endothelial cell dysfunction in numerous diseases.

Disease models	Disease models	Compound	Chemical classification	Effects	Mechanism of action	Reference
Natural products						
AD	APP/PS1 mice, Aβ_25‐35_ induced bEnd.3	Lychee seed polyphenols	Polyphenols	Improve BBB integrity and cognitive function. TJ upregulation	Autophagy pathway (ASC/LC3/AMPK/mTOR/ULK1) and pyroptosis pathway (NLRP3/Caspase 1/IL‐1β)	[245]
AD	5 × FAD mice	Crocus sativus extract	Natural compound mixture	Tighten cell‐based BBB model (IgG extravasation and Claudin‐5 expression) and reduce Aβ burden and neuroinflammation	Enhance Aβ clearance through BBB (ABCB1 [P‐gp], LRP1, ABCA1), enzymatic degradation (IDE, NEP) and ApoE clearance (ABCA1, ApoE, PPARγ) Anti‐inflammatory and anti‐oxidative compounds	[14]
AD	Aβ_1‐42_ induced bEnd.3	Hyperoside	Flavanol glycoside	Reverse the expression of ZO‐1, Claudin‐5, and Occludin and decrease MMP‐2 and MMP‐9	Suppress mitochondrial apoptosis as indicated by ratios of Bax/Bcl‐2, cleaved caspase‐9 (8, 12)/caspase‐9 (8, 12), as well as cytochrome c and caspase‐3 activity	[117]
AD	Aβ_1‐42_ induced bEnd.3	Catapol	Iridoid glucoside	Decrease permeability, endothelial apoptosis and Increase ZO‐1, Claudin‐5, Occludin and LRP1, ABCB1 (P‐gp). Aβ efflux is enhances	Decrease MMP2 and MMP9, inhibit RAGE. Mitochondrial and death receptor mediated apoptosis pathway is inhibited	[118]
AD	Aβ_1‐42_ induced bEnd.3 cells	EGb761, standard Ginkgo biloba extract	Natural compound mixture	Decrease BBB permeability and increase ZO‐1, Claudin‐5, and Occludin	Decrease RAGE and ROS	[226]
AD	Aβ_25‐35_ induced bEnd.3	Lutein	Xanthophylls	Increase endothelial cell survival and decrease oxidative stress	Inhibit NFκB pathway and mitochondrial apoptosis pathway and activate Nrf2 pathway	[123]
scienAD	TgSwDI mice	Oleocanthal	Phenolic compound	Increase Amyloid‐beta clearance	Increase ABCB1 (P‐gp), LRP‐1 and APOE mediated clearance pathway and decrease IL‐1β	[177]
AD	APP/PS1 mice	Pinocembrin	Flavonoids	Maintain neuropil ultrastructure and microvascular function, reduce glial reduction, inflammatory mediators	Inhibit RAGE‐mediated transduction	[122]
AD	Aβ_1‐42_ induced hBMECs	Asiaticoside	Trisaccharide triterpene	Improve cell survival and restore mitochondrial membrane potential	Decrease TLR4, MyD88, TRAF6, p‐NFκB p65 and p65 nuclear translocation	[205]
AD	Aβ_1‐42_ induced HBMEC cells	Zhenxin Xingshui Yizhi Fang	Chinese herbal formulation	Upregulate LRP1, GLUT1, and GLUT3	Inhibit caspase 3 mediate apoptosis and downregulate RAGE	[239]
AD	APP/PS1 mice	Omega‐3 polyunsaturated fatty acids	Fatty acids	Increase LRP‐1 and promote Aβ clearance	Inhibit NFκB pathway, reduce IL‐1β, TNFα and suppress glial activation	[248]
Nucleic acid therapeutics						
AD	PS19 tau‐Tg mice	C3R−/− knockdown	Gene‐knockdown	Decrease VCAM‐1 and increase brain vessel cross‐section area	Inhibit C3R mediated vascular inflammation and barrier integrity and microglial activation	[174]
AD	TgCRND8 mice (K670N, M671L and V717F)	FXII‐ASO (antisense oligonucleotide)	RNA knockdown	Reduce neuroinflammation, fibrinogen deposition, neurodegeneration, and improved cognitive function	Inhibit FXII‐mediated contact system activation	[35]
AD	APP/PS1	siR/PIO@RP	RNA silencing and agonist	Downregulate RAGE and activate PPARγ	Inhibit RAGE‐mediated neuroinflammation, repair NVU injury and facilitate LRP‐1‐mediated Aβ clearance Activate PPARγ‐mediated neurotrophic effects	[241]
Proteins, peptides, or enzymes						
AD	5 × FAD mice and Aβ_1‐42_ induced bEnd.3 cells	Human recombinant annexin A1	Protein	Upregulate ZO‐1 and Claudin‐5 and BBB integrity. pericyte‐derived annexin‐A1 also recover BBB integrity	Inhibit RhoA‐ROCK singling pathway. Anti‐inflammatory messenger	[162]
AD	5 × FAD mice Aβ‐induced bEnd.3 cells	Acrp30	A globular form adiponectin	Reduce proinflammatory cytokines and RAGE. Endothelial reverse apoptosis, TJ disruption in Aβ‐induced bEnd.3	Suppressed inflammatory signaling through AdipoR1‐mediated NFκB	[206]
AD	APP/PS1	TNFI, TNF inhibitor (cTfRMAb‐TNFR), engineered by the fusion of the extracellular domain of the type II human TNF receptor (TNFR) to a chimeric monoclonal antibody (mAb) against the mouse transferrin receptor (TfR)	Protein	Reduce brain Aβ burden, neuroinflammatory markers, and parenchymal IgG, as well as improve cognitive function	Inhibit TNF effect	[31]
AD	ApoE4‐targeted replacement mice	CS‐6253, a peptide can activate ABCA1	Peptide	Reversal of Aβ_1‐42_ accumulation and tau hyperphosphorylation, as well as cognitive deficits and synaptic impairment	Activate ABCA1, increased ApoE lipidation	[24]
AD	Aβ_1‐42_ induced hCMEC/D3	Somatostatin	Peptide	Improve TJ proteins and regulate LRP1 and RAGE expression and increased Aβ reuptake	Abrogate Aβ‐induced JNK phosphorylation and expression of IL‐1β and MMP‐2	[159]
AD	Homozygous 3 × FAD mice	L‐norvaline	Amino acid	Reduce BBB permeability, amyloid angiopathy, microgliosis, astrodegeneration	Alleviate astrocyte and microglia pathology	[169]
AD	Aβ_25‐35_ induced bEnd.3	CoQ10	Enzyme	Prevent apoptosis and necrosis	Inhibit NAPDH oxidase activity and reduce ROS and Ca^2+^. CoQ10 inhibits the entry of Aβ_25‐35_ into mitochondrial.	[60]
Approved Drugs						
AD	TgCRND8 mice	Dabigatran	Anticoagulant drug	Prevent memory decline, cerebral hypoperfusion, toxic fibrin deposition, T cells infiltration. AQP4 at astrocyte endfeet is maintained. AD related astrogliosis, pericyte alteration is also alleviated.	Anti‐thrombin, anticoagulation, anti‐inflammatory effect, decrease fibrinogen.	[41]
AD	Aβ_1‐42_ induced bEnd.3 cells	Azelnidipine	Calcium channel blocker drug	Reduced superoxide anion production	Inhibit ERK1/2 activation, cPLA2 phosphorylation and NFκB activation	[218]
Others						
AD	Homocysteine‐induced AD	Hydrogen sulfide	Chemical compound	Decrease microvascular permeability and upregulate claudin‐5, VE‐Cadherin	Decrease MMP‐2, MMP‐9, ICAM‐1, GFAP, BDNF, synaptosomal Ca^2+^ and synaptic functional proteins	[89]
AD	Tg2576 mice	High fat diet	Diet therapy	Improve learning function, decrease BBB leakage, and ventricles volume	Lipid metabolism, brain insulin signaling. Insulin receptor is increased in hippocampus	[53]
Natural products						
Ischemia stroke	OGD/R induced bEnd.3 cells	Acetyl‐11‐keto‐β‐boswellic acid	Triterpenoid compound, a novel Nrf2 activator	Upregulate ZO‐1 and occludin Preserve endothelial survival	Attenuate inflammation and oxidative stress Increased phosphorylation of ERK	[1]
Ischemia stroke	Chronic hypoxia and hypoglycemic condition (CHH)	EGb761, standard Ginkgo biloba extract	Natural compound mixture	Increase LRP‐1 and decrease RAGE	—	[247]
Ischemia stroke	tMCAO/R	Quercetin	Flavonoids	Decrease BBB permeability and ROS generation	Activate Sirt1/Nrf/HO‐1 signaling	[251]
Ischemia stroke	Ischemia reperfusion induced rat	Kaempferol	Flavonoids	Attenuate BBB disruption and inflammation	Decrease NFκB p65 and nuclear translocation	[112]
Ischemia stroke	OGD/R induced endothelial‐astrocyte coculture system	Geniposide	Iridoid glycoside	Decrease BBB permeability, increase TJ protein (ZO‐1, Claudin‐5, Occludin)	Decrease MMP‐9, MMP‐2, inflammatory cytokines, increase BDNF, and astrocyte derived neurotrophic factor.	[109]
Ischemia stroke	MCAO model	Tetramethylpyrazine (TMP)	Alkaloids	Decrease BBB permeability, increase TJ proteins	Inhibit the activation of JAK2/STAT3 signaling pathway.	[68]
Ischemia stroke	MCAO wistar rats	Alpha‐pinene	Terpene	Decrease BBB permeability	Decrease TNF‐α and IL‐1β, suppress apoptosis as indicated by the ration of Bax/Bcl‐2	[96]
Ischemia stroke	MCAO wistar rats	Morin	Flavonoids	Decrease BBB permeability, neutrophil infiltration, cerebral damage, and increase TJ proteins	Attenuate inflammation and ROS production via reducing TLR4, NFκB	[94]
Ischemia stroke	OGD‐induced cell models	Myricetin	Flavonoids	Decrease endothelial permeability, oxidative stress, inflammation	Increase NO production by upregulating peNOS (S1177)/NO pathway and stimulate eNOS coupling and activity in a Nrf2/Akt‐dependent manner	[78]
Ischemia stroke	MCAO SD rats and OGD‐induced BMECs	Catalpol	Iridoid glucoside	Reduce neurological deficit, infarct volume, and protect vascular structure and promote angiogenesis	Activate HIF‐1α/VEGF pathway	[235]
Ischemia stroke	MCAO mice and OGD‐induced bEnd.3	Medioresionol	Furofuran type lignan	Reduce brain infarct and BBB permeability, inhibite pyroptosis, promote neurobehavioral function, and TJ expression	ameliorate pyoptosis (NLRP3/ASC/Cleaved caspase 1, IL‐1β, GSDMD‐NT), and mtROS by promote interaction of PGC‐1α and PPARα and increase PPARα nuclear translocation, further increase PAH and GOT1 to inhibt excess accumulation of phenylalanine induced mtROS and pyroptosis pathway	[234]
Ischemia stroke	OGD/R‐induced cell model	Salvianolate lyophilized and Xueshuantong injection	Herbal standardized preparations	Increase TEER and decrease permeability and enhance TJ proteins	Increase Ang‐1 and Tie‐2 but decrease Ang‐2 and VEGF.	[258]
Ischemia stroke	OGD/R induced bEnd.3 cells and SD rats with spinal cord injury	17β‐estradiol (E2)	Steroid hormone	Improve the TJ junctions and decreased brain spinal cord barrier injury	Suppress NFκB pathway and subsequent MMP‐1b, MMP‐2, MMP‐3, MMP‐9, MMP‐10, MMP‐13 through recruitment of ERα	[149]
Nucleic acid therapeutics						
Ischemia stroke	MCAO	EC‐targeted overexpression of Krupple‐like factor 11 (KLF11)	Gene overexpression	Decrease BBB leakage and proinflammatory factors, preserve TJ levels	PPARγ/KLF11	(Zhang, [271])
Ischemia stroke	MCAO mice and OGD‐induced mBMECs	Ablation of miR‐15a/16–2 microRNA cluster	RNA knockdown	Prevent BBB breakdown and infiltration of peripheral immune cells	Inhibit miR‐15a/16–1 targeted‐inhibition of Claudin‐5 gene expression	[129]
Subarachnoid hemorrhage	SAH SD rats	Mfsd2a upregulation	Gene overexpression	Reverse BBB damage	Inhibit caveolae‐based transcellular transport by transporting omega‐3 fatty acids to protect BBB	[273]
Intracerebral hemorrhage	ICH rat model and BMECs	miR‐126‐3p	miRNA	Attenuate BBB disruption	miR‐126‐3p target VCAM‐1 gene and downregulate its expression	[62]
Intracerebral hemorrhage	ICH mice and BMECs	Blnc1 siRNA	siRNA	Decrease permeability, apoptosis, inflammation, migration	Suppress PPARγ/SIRT6‐mediated FoxO3 activation	[243]
Approved Drugs						
Ischemia stroke	Hypoxia induced HBMECs	Propofol	Intravenous anesthetic drug	Increase BBB integrity, upregulate ZO‐1 and decrease ZO‐1 phosphorylation	Probably inhibit HIF‐1α, VEGF, and CaMKII, as well as chelate Ca^2+^ uprsie	[33]
Ischemia stroke	MCAO/R rats	Dexamethasone, YAP agonist	Chemical compound	Improve neurological function, smaller brain infarct, TJ protein expression and decrease BBB permeability	Activate Hippo/YAP/TAZ signaling	[33]
Intracerebral hemorrhage	Collagenase‐induced ICH model	Minocycline	Antibiotic drug	Decreased BBB disruption and neurological deficits	Decrease production of proinflammatory mediators and decrease DKK expression but increase Wnt1, β‐catenin and Occludin	[228]
Chemical compounds						
Ischemia stroke	MCAO model, hypoxia induced bEnd.3	Agomelatine, melatonin receptor agonist	Agonist of melatonin	Upregulate claudin‐5 in the cerebral cortex and decrease macrophage infiltration	CD68, MCP‐1 is downregulated Anti‐inflammatory and anti‐oxidative compounds	[27]
Ischemia stroke	MCAO/R	Lithium	Chemical element	Increase BBB integrity, Increased Claudin‐5 and ZO‐1, decrease MMP‐9	Upregulate the activity of endothelial Wnt/β‐catenin signaling mediated by Gpr124	[86]
Ischemia stroke	MCAO and OGD‐induced cell models	Lithium	Chemical element	DecMPrease BBB permeability, inhibit MMP‐9 activity, and neutrophil invasion and increase T cell extravasation	Activate MAPK/ERK1/2 pathway	[74]
Ischemia stroke	Ischemia reperfusion induced rat	Magnesium sulfate	Chemical compound	Decreased BBB permeability	Reduce lipid peroxidation and increase antioxidant enzymes	[197]
Ischemia stroke	MCAO rats	TGR5 agonist, INT777	Chemical compound	Decrease BBB permeability and increase TJ proteins	Upregulate the expression of BRCA1 and Sirt1	[113]
Ischemia stroke	OGD/R induced bEnd.3 cells	2,4,5‐trihydroxybenzaldehyde (TDB)	Chemical compound	Endothelial apoptosis	Suppress miR‐34a, therefore upregulate Bcl‐2 and suppress caspase‐9/3 pathway. Maintain mitochondrial membrane potential (MMP).	[114]
Subarachnoid hemorrhage	SAH SD rats	Mitoquinone	Mitochondrial target antioxidant	Attenuate brain edema, increase Claudin‐5	Nrf2/PHB2/OPA1 pathway	[269]
Cellular therapy						
Ischemia stroke	tMCAO and OGD‐induced bEnd.3	Intracranial injection of mesenchymal stem cells (MSCs)	Cellular therapy	Reduce infarct volume, improve behavioral function, IgG leakage, TJ loss, inflammatory cytokines, MMP‐9 expression, and activity	AMPK and ICAM are involved in the modulatory effects of MSCs on MMP‐9	[37]
Ischemia stroke	MCAO mice and OGD‐induced bEnd.3	Transplantation of oligodendrocyte precursor cells	Cellular therapy	Decrease BBB leakage and increase Claudin‐5 and β catenin	Effect is similar to Wnt7a, which increases β catenin and Claudin‐5 Activating Wnt/β catenin pathway	[231]
Others						
Ischemia stroke	Rat thromboembolic stroke model	Remote ischemic conditioning (RIC)	Therapeutic intervention	Reduce BBB injury, intracerebral hemorrhage, cerebral infarction, and neurological deficits	Reduce PDGF‐CC/PDGFRα pathway	[75]
Natural products						
Inflammation	TNF‐α‐induced EC	Honokiol	Biphenolic phytochemical compound	Neutrophil adhesion	Decrease VCAM‐1 and inhibiting ubiquitination‐mediated IκBα degradation and NFκB nuclear translocation	[77]
Inflammation	LPS‐induced bEnd.3 cells	*Panax notoginseng* saponins (PNS)	Mixture of saponins	Increase TEER and decrease permeability. Upregulate TJ proteins	Anti‐inflammatory effect and anti‐oxidative effect (PI3K/Akt/NRF2 or NFκB)	[78]
Inflammation	LPS‐induced bEnd.3 cells	Chrysin (5,7‐dihydroxyflavone)	Flavonoids	Decrease leukocyte adhesion	Inhibit MAPK and NFκB pathway and decrease VCAM‐1	[104]
Inflammation	TNFα‐induced mice	Secoisolariciresinol diglucoside	Phytoestrogen	Decrease leukocyte adhesion and migration, BBB permeability in vivo. In vitro found decreased monocyte adhesion and migration	Decrease the expression of VCAM‐1 and VLA‐4 integrin, which are needed for leukocyte infiltration	[186]
Inflammation	LPS‐induced striatum injury in mice	Kaempferol	Flavonoids	Maintain BBB integrity, decrease proinflammatory cytokines, adhesion molecules, COX‐2	Inactivate HMGB1/TLR4	[252]
Inflammation	LPS‐induced mice and HBMECs	*trans*‐10‐hydroxy‐2‐decenoic acid (10‐HDA)	Acid	Decrease BBB permeability, inhibit the degradation of TJ proteins	Inhibit proinflammatory mediators, cytokines, chemokines, adhesion molecules, and MMP‐2 and MMP‐9 Promote activation of AMPK/PI3K/AKT	[256]
Inflammation	LPS‐induced mice and bEnd.3 cells	17β‐estradiol (E2)	Steroid hormone	Decrease monocyte adhesion and expression of acute‐phase proteins	Anti‐inflammatory effect	[65]
EAE	EAE mice	Vitamin D (1,25‐dihydroxyvitamin D3)	Vitamin	Decrease blood spinal cord barrier permeability	Decrease neuroinflammation (NLRP3, caspase‐1, IL‐1β, CX3CR1, CCL17, RORc, and Tbx21)	[46]
Inflammation	IL‐1β‐induced pericyte coculture with ECs	Melatonin	Hormone	Improve BBB integrity and upregulate ZO‐1, Claudin‐5, Occludin, and VE‐Cadherin	Decrease pericyte secretion of MMP‐9 and upregulate TIMP‐1 via NOTCH3/NFκB	[176]
Inflammation	LPS‐induced mice	Stearoylethanolamide	Endocannabinoid neurotransmitter	Support BBB integrity	Restrict peripheral inflammation and leukocyte infiltration and inhibit microglial activation by cannabinoid receptors CB_1/2_	[90]
Inflammation	LPS‐induced mice	Fenretinide	Synthetic retinoid derivative	Decrease BBB permeability	Nrf2 and NFκB pathway	[111]
Oxidative stress	H_2_O_2_‐induced bEnd.3 cells	Glutathione	Antioxidant in nature body	Decreased NO, ROS, and improve TJ junction proteins	Activate Nrf2 pathway	[207]
Chemical compounds						
EAE	EAE mice	CGS‐21680, A2A receptor specific agonist	Small molecule compound	Reverse TJ protein expression, decrease permeability, and neurologic deficiency	Prevent Th1 stimulation and MLCK phosphorylation signaling	[124]
Oxidative stress	H_2_O_2_‐induced bEnd.3 cells	Nicotinamide mononucleotide	Nucleotide derived from ribose	Prevent against apoptosis and inflammation	Inhibit NFκB p65 and increase enzyme NAMPT, VEGF, and eNOS against apoptosis	[48]
Multiple sclerosis	TNFα and IFNγ induced in vitro BBB model	Siponimod, approved for MS treatment by targeting S1P1 and S1P5	Small molecule drug	Upregulate ZO‐1 and Claudin‐5, decrease MMP‐9	Activate PI3K/Akt pathway.	[208]
Cellular therapy						
Inflammation	LPS‐treated rats	Mesenchymal stem cells (MSCs)	Cellular therapy	Restore BBB permeability and endothelial barrier antigen and ABCB1 (P‐gp) expressing cells. Increase astrocyte filament around endothelial cell and VEGF‐A and eNOS elevations	Suppress VEGF‐A and VEGF‐A induced VEGFR2/eNOS activation and subsequent downregulation of TJ proteins. Suppress LPS‐induced secretion of IL‐1β and VEGF‐A, respectively in microglia and astrocytes.	[161]
Natural products						
Type‐2 Diabetes		Cannabinoids	Natural compound mixture	Reverse BBB damage	Anti‐inflammatory, anti‐apoptotic effects and upregulate TJ proteins	[25]
Traumatic brain injury	TBI mice	Ginsenoside Rg1	Steroidal saponin	Enhance TIMP3 expression and MMPs proteolysis, restrict TJ protein degradations, and improve BBB integrity Suppress apoptosis and NFκB mediated inflammation, increase GFAP, and VEGF	Inhibit exos‐miR‐21 release in peripheral blood flow to brain	[266]
Traumatic brain injury	TBI mice	Ghrelin	Hormone	Restore vascular permeability, decrease apoptosis.	Increase UCP‐2 and decrease caspase‐3	[127]
Traumatic brain injury	TBI mice model	All‐trans retinoic acid	Retinoic acid	Decrease BBB permeability	Decrease HMGB1, mitochondrial apoptosis marker, microglial activation marker (TSPO), astrogliosis maker (GFAP, Serpina3n)	[83]
Approved drugs						
Type‐2 Diabetes	High‐fat and high fructose‐fed mice	Probucol	Antihyperlipidemic drug	Improve BBB integrity	Anti‐inflammatory and anti‐oxidative effects	[131]
Chemical Compounds						
Type‐2 Diabetes	High‐fat diet induced mice	Adenosine receptor 2a (Adora2a) antagonist	Chemical compound	Improve BBB integrity, TJ protein expression	Decrease vascular inflammation	[246]
Traumatic brain injury	TBI mice	DI‐3n‐butylphthalide	Chemical compound	Upregulate TJ proteins and improve neuronal survival	Decrease ATG7/Beclin1/LC3II and mitochondrial apoptosis, as well as anti‐inflammatory, anti‐oxidative, anti‐apoptotic, and mitochondrion‐protective functions.	[237]
Non treated		Apolipoprotein M‐bound sphingosine 1‐phosphate (S1P) or S1PR1 agonist SEW2871	Signaling sphingolipid	Maintain paracellular BBB permeability for small molecules in all cerebral microvessels and low levels of vesicle‐mediated transport in penetrating arterioles	S1PR1 stimulation inhibits neuroinflammation and maintain BBB integrity	[136]
Non treated	hCMEC/D3	Exogenous Wnt ligand (Wnt3a) or LiCl (GSK inhibitor)	Chemical compound	Reduce permeability, Increase the activity of ABCB1 (P‐gp) and BCRP	Activation of Wnt/β catenin pathway	[101]

### 5.2 Vascular Inflammation and Oxidative Stress

#### 5.2.1 Signaling Pathway

Vascular inflammation is an important factor causing BBB damage and can also induce abnormality of junctional proteins and transporters. Most of natural herbal products exert anti‐inflammatory and anti‐oxidative effects. Inhibition of TLR4/NFκB is generally adopted strategies for the BBB protective effects of these herbal products in treating AD [123]. In AD, RAGE is the predominant receptor mediating Aβ pathology. Inhibition of RAGE‐mediated NFκB activation exerts BBB protective effects in AD model and Aβ‐mediated pathology [122].

The therapeutic effect of NFκB inhition is also reported in other neurodegenerative diseases like ischemia stroke [112]. Inhibition of TLR4/NFκB by Morin and inactivation of HMGB1/TLR4 by Kaempferol protect BBB in MCAO rats [94] and mice with LPS‐induced striatum injury [252].

Oxidative stress contributes to BBB leakage. Attenuating Aβ‐induced oxidative stress restores endothelial survival. It is found that antioxidant CoQ10 inhibits the entry of Aβ into mitochondrial and the hyperactivity of NAPDH oxidase, and overproduction of Ca^2+^ and ROS, eventually prevents EC apoptosis and necrosis [60]. Decreasing Ca^2+^ is proved to have BBB protective effect. Calcium channel blocker drug, Azelnidipine protects BBB against Aβ stimulation which involve the inhibition of superoxide anion overproduction and the inhibition of ERK/cPLA2/NFκB pathway is also involved [218]. Therefore, it indicates that Ca^2+^ uprise, NFκB activation and oxidative stress coexist under Aβ pathology. In ischemic stroke model, the antioxidant protein Nrf2 is shown as a potential target for BBB protection. Nrf2 activator, Acetyl‐11‐keto‐β‐boswellic acid upregulates TJ proteins and maintains EC survival against OGD stimulation [1]. Increased ERK phosphorylation and activation of HO‐1/Sirt1 are involved in Nrf2‐mediated BBB protective effects respectively reported in OGD model [1] and tMCAO mice [251]. Moreover, in hemorrhage rat model, Nrf2/PHB2/OPA1 pathway is related to the attenuation of brain edema and upregulation of claudin‐5 after the treatment of Mitoquinone [268]. In addition to NFκB and Nrf2, IL‐6R/gp130/JAK2/STAT3 pathway commonly mediates proinflammatory effect of IL‐6 and is involved in BBB impairment. In MCAO mice, inhibition of JAK2/STAT3 by tetramethylpyrazine (TMP) protects BBB integrity and upregulates TJ proteins [69].

#### 5.2.2 Receptors and Ligands

Compounds by modifying receptors or ligands that mediate the vascular inflammation have shown BBB protective effects. C3a receptor (C3aR) activation participates in triggering VCAM‐1 mediated CD8^+^ T cell infiltration and Ca^2+^ elevation‐mediated reorganization of cadherin junction and cytoskeleton, as well as microglial activation. In Tau301S‐Tg mice, knockdown of C3aR attenuates VCAM‐1‐mediated vascular inflammation and microglial inflammation [174]. Cannabinoid receptors CB_1/2_ is reported as a potential target for BBB protection upon LPS stimuli. Stearoyl ethanolamide attenuates vascular inflammation and microglial activation in LPS‐induced mice model and the activation of CB_1/2_ is involved [90]. Similar finding is also reported in that cannabinoids treatment reverses BBB damage in type‐2 diabetes [25]. Adenosine A2A receptor in ECs closely relates to vascular inflammation [125]. CGS‐2180, a specific A2A receptor agonist protects BBB integrity against the stimulation of Th1 cells in EAE mice and the suppression of MLCK signaling is involved [124]. However, the pathological effect of A2A signaling differs according to disease conditions. In high‐fat diet induced type‐2 diabetes model, A2A receptor antagonism in turn improves BBB integrity and upregulates TJ proteins [246]. In ischemic mice, inactivation of A2A receptor protects the brain injury [279]. Shingosine 1 phosphate (S1P) protects BBB by inhibiting inflammation. The treatment of Apolipoprotein M‐bound S1P or S1P receptor 1 (S1P1) agonist SEW2871 maintain BBB permeability in ApoM‐deficient mice model (Mathiesen [136]). The protective role of S1P1 is also evidenced by an approved drug Siponimod which has affinity to S1P1 and S1P5 that protect BBB integrity in MS mcie. Siponimod upregulates ZO‐1 and Claudin‐5 by activating S1P1 and decrease MMP‐9 in TNFα‐ and IFNγ‐induced ECs by activating S1P5, in which the activation of PI3K/AKT is involved [208]. Activation of AMPK/PI3K/AKT is also involved in the anti‐inflammatory effects of trans‐10‐hydroxy‐2‐decenoic acid (10‐HDA) on LPS‐induced ECs [256]. Numerous studies have demonstrated the potentials of adiponectin and adiponectin receptor in ameliorating brain injury and glial inflammation [233]. In 5 × FAD mice and Aβ‐induced ECs, treatment of a globular form adiponectin (Acrp30) suppresses activation of RAGE/NFκB and endothelial apoptosis, meanwhile upregulates TJ proteins [206]. Proinflammatory cytokines dissociate BBB integrity and disturb transporter activity. Reducing the levels of cytokines show protective effect on BBB. For example, TNF inhibitor constructed by fusing the extracellular domain of TNF receptor with antibody against transferrin receptor improves BBB integrity and cognitive functions in APP/PS1 mice, meanwhile, the Aβ burden and neuroinflammation are also attenuated [31].

#### 5.2.3 Approved Drugs

As mentioned above, factor XII‐induced contact‐system activation contributes to vascular inflammation. Knockdown of Factor XII mRNA by antisense oligonucleotide attenuates fibrinogen deposition and neuroinflammation [35]. Anticoagulant drug, dabigatran also shows BBB protective effects in TgCRND8 mice and inhibits inflammation‐, thrombin‐, fibrinogen‐mediated toxicity on ECs. Especially, Dabigatran maintains AQP4 protein levels at astrocytic endfeet, which is essential for structural coupling between astrocyte and endothelial cell. In addition, it inhibits astrogliosis and pericyte degeneration in TgCRND8 mice [41]. Therefore, it suggests that dabigatran is a promising drug for BBB protection. Another approved antihyperlipidemic drug probucol also exerts BBB protective effects in high‐fat diet induced type 2 diabetes model by attenuating inflammation and oxidative stress [131].

### 5.3 Autophagy Pathway

Contradictory findings on autophagy are suggested above, which is enhanced in Aβ‐induced ECs [30], however impaired in iPSCs (extracted from FAD patients)‐derived ECs with PSEN mutations [180]. Increased autophagy in Aβ‐induced EC maybe a defensive event for enhanced Aβ degradation and clearance. Enhanced autophagy involves decreased phosphorylation of mTOR. In hAPP (J20) mice and LDLR^−/−^ mice, which respectively mimic AD and vascular dementia, inhibiting mTOR by rapamycin ameliorates BBB breakdown manifested as upregulated TJ proteins and attenuated vascular inflammation (Van [220]). Moreover, inhibition of mTOR reduces ROS and MMP‐9, as well as activates protective eNOS/NO. Therefore, it may indicate the therapeutic potential of mTOR inhibition and enhanced autophagy in BBB protection. However, decreased phosphorylation of mTOR may result in inhibited autophagy. Therefore, more evidences are required to clarify the relationship among BBB integrity, autophagy, and mTOR phosphorylation. Inhibiting autophagy pathway (ATG7/Beclin1/LC3II) by DI‐3n‐butylphthalide also upregulates junctional proteins (Occludin, β catenin) in TBI mice [238]. However, contradictory finding is reported in 3 × Tg‐AD mice that enhanced autophagy correlates with downregulation of TJ protein [30] and transporters (LRP‐1, ABCB1 [P‐gp]) [64]. Treatment of lychee seed polyphenols inhibits autophagy pathway (ASC/LC3/AMPK/mTOR/ULK1) and upregulates TJ proteins in AD animal model and Aβ‐induced ECs [245]. No matter the enhancement or inhibition of autophagy pathway, inhibition of mTOR always show therapeutic potentials on BBB.

### 5.4 Mitochondrial Apoptosis, Death Receptor Mediated Apoptosis and Pyroptosis

BBB breakdown is partially caused by EC death. Mitochondrial or death receptor mediated apoptosis and pyroptosis can lead to EC death. As suggested above, mitochondrial apoptosis and pyroptosis occur in numerous AD models. Suppression of mitochondrial apoptosis by hyperoside maintains endothelial integrity in Aβ‐induced ECs by upregulating TJ proteins and decreasing MMPs [117]. Inhibition of mitochondrial apoptosis or death receptor mediated apoptosis by catapol [118] and Zhenxin Xingshui Yizhi Fang [239] enhances Aβ efflux and glucose influx in Aβ‐induced ECs respectively due to upregulated efflux transporters (LRP‐1 and ABCB1 [P‐gp]), and influx transporters (GLUT1 and GLUT3). Asiaticoside, belonging to trisaccharide triterpene, reversed Aβ‐induced endothelial apoptosis and mitochondrial membrane potential, the underlying mechanism of which also involves inhibition of TLR4/MyD88/TRAF6/NFκB [205]. In other neurodegenerative diseases, inhibition of apoptosis also acts as a potential way for BBB protection. Inhibition of mitochondrial apoptosis protects BBB in MCAO rats (alpha‐pinene) [96] and attenuates inflammation in TBI mice (Ghrelin) [127].

Activation of pyroptosis pathway including the activation of NLRP3/Caspase‐1/IL‐1β is observed in APP/PS1 models and Aβ‐induced ECs. Inhibition of pyroptosis is involved in EC protective effects of lychee seed polyphenols in upregulating TJ expression and improving BBB integrity as well as cognitive function [245]. In ischemic stroke, medioresionol, belonging to Furofuran type lignan also protects BBB integrity in MCAO and OGD‐induced ECs by activating upstream signaling for pyroptosis including PGC‐1α/PPARα/PAH/GOT1, further inhibiting the overproduction of mitochondrial ROS and pyroptosis pathway (NLRP3/ASC/Caspase‐1/IL‐1β) [234].

### 5.5 Proangiogenic Pathway

Proangiogenic pathway mediates angiogenesis. Growth factors such as VEGF and angiopoietu (Ang) can trigger angiogenesis by activating proangiogenic pathway. BBB permeability is increased during angiogenesis. VEGF‐A produced by BMECs or astrocytes is known as a key factor for BBB breakdown through activating VEGFR2‐mediated proangiogenic pathway, which eventually results in eNOS‐mediated TJ downregulation [6]. Transplantation of mesenchymal stem cells (MSCs) to LPS‐induced rats restores BBB integrity by reducing VEGF‐A level and suppressing VEGF‐A/VEGFR2/eNOS activation. MSCs transplantation downregulates VEGF‐A mainly by reducing its secretion from astrocytes and IL‐1β secretion by microglia, which could further stimulate the production of VEGF‐A from astrocytes [161]. However, phosphorylation of eNOS at S1177 is beneficial from endothelial function by improving NO production. For instance, myricetin protects endothelial integrity and attenuates inflammation by boosting eNOS/NO pathway via Nrf2/Akt‐dependent manner in OGD‐induced cell model [78]. Whereas, eNOS/NO uncoupling leads to decreased production of NO and ROS overproduction, thus contributes to BBB breakdown. The protective effect of myricetin also include maintaining eNOS/NO coupling and thus promoting the production of protective NO. The production of VEGF in BMECs depends on the activation of HIF1α. Inhibition of HIF‐1α/VEGF, along with Ca^2+^/CaMKII by propofol protects BBB integrity and upregulates ZO‐1 [33]. Findings about the protective role of proangiogenic pathway are also reported. Study shows that activation of endothelial HIF‐1α/VEGF pathway by catalpol protects vascular structure and neurological functions in MCAO and OGD‐induced ECs [229]. In H_2_O_2_‐induced EC model, increase of VEGF and eNOS are also involved in the anti‐inflammatory and anti‐apoptotic effects of nicotinamide mononucleotide, which mainly exerts protection by upregulating the cardiovascular protective enzyme NAMPT [48]. Similarly, activation of ANG‐1/Tie‐2 is involved in the effects of BBB protection and TJ upregulation by salvianolate lyophilized and xueshuantong injection in OGD/R‐induced cell model, accompanied with decrease of Ang‐2 and VEGF [258]. We also found that suppression of VEGF signaling is involved in Aβ oligomer or Aβ oligomer‐induced astrocyte secretion induced BEC dysfunctions [261]. Therefore, more investigations are still in need to elucidate the therapeutic strategies by targeting VEGF.

### 5.6 Wnt/β‐Catenin Pathway

Wnt/β‐catenin pathway regulates the expression of junctional proteins in ECs. Treatment of exogenous Wnt ligand, Wnt3a decreases BBB permeability and increases the activity of ABCB1 (P‐gp), and BCRP by activating Wnt/β‐catenin pathway [101, 135]. Upregulation of Wnt and β‐catenin is involved in BBB protective effects of minocycline in collagenase‐induced ICH model, accompanied with upregulated occludin and decreased production of proinflammatory mediators [228]. Activation of Wnt/β‐catenin signaling mediated by Gpr124 also accounts for the protective effect of lithium in upregulating ZO‐1, claudin‐5, and decreasing MMP9 in MCAO/R model [86]. In addition, the protective effect of lithium on BBB integrity and vascular inflammation in MCAO/R animal model also involves the activation of MAPK/ERK1/2 [74]. Oligodendrocyte precursor cells (OPCs) are involved in maintaining BBB integrity. Study shows that activation of Wnt/β‐catenin signaling is responsible for the protective effects of transplantation of oligodendrocyte precursor cells on MCAO mouse, in which BBB integrity is improved with claudin‐5 upregulated. Furthermore, Wnt7a treatment upregulate the expression of claudin‐5 and β‐catenin in OGD‐induced ECs [231].

### 5.7 Matrix Metalloproteinases

MMPs can digest TJ and basement membrane proteins, thus contributing to BBB leakage [182]. Astrocytes and pericytes secrete excessive MMPs under disease conditions, acting as extracellular stimuli for BBB disruption. In ECs, activation of NFκB signaling is responsible for MMPs upregulation. In OGD‐induced cell model and SD rats with spinal cord injury, 17β‐estradiol (E2) downregulates a series of MMPs, including MMP‐1b, MMP‐2, MMP‐3, MMP‐9, MMP‐10, MMP‐13, accompanied with upregulation of TJ proteins and the suppression of NFκB pathway via recruitment of estrogen receptor α is involved [149]. Intracranial injection of mesenchymal stem cells (MSCs) downregulates MMP‐9 expression and inhibits its activity, eventually improving the BBB integrity and attenuating neuroinflammation. Meanwhile, the downregulation of AMPK and ICAM‐1 are involved in this process [37]. Study also indicates the protective role of tissue inhibitor of metalloproteinase‐1 (TIMP1) in BBB protection, which can proteolyze MMPs. It is found that recombinant TIMP‐1 improves TJ proteins and transendotheial tightness and the activation of FAK signaling, suppression of RhoA, and F‐actin depolymerization are involved [217]. TIMP3 may also be a potential target to decrease MMPs level. A study reported that ginsenoside Rg1 could enhance TIMP3 and MMPs proteolyzation, therefore protect BBB integrity in TBI mice [266].

### 5.8 Aβ Clearance Pathway

Enhancing Aβ clearance via BBB is always the important goal for AD therapy. Main strategies include increasing Aβ clearance through efflux transporter ABCB1 (P‐gp)/LRP‐1‐mediated pathway and APOE‐mediated clearance pathway (ABCA1/APOE/PPARγ) [177], as well as downregulating influx transporter RAGE. Inhibition of vascular inflammation [248] and mitochondrial apoptosis [118] could also increase Aβ clearance and upregulation of related transporters. Specifically, in hAPP mice, blocking ABCB1 (P‐gp) internalization by nacodazole, a microtubule inhibitor maintains ABCB1 (P‐gp) level on membrane and enhances Aβ transport through BBB, reduces Aβ_1‐40_ and Aβ_1‐42_ in brain capillary brain [50]. APOE lipidation is required for Aβ clearance through LRP‐1. Hypolipidation of APOE4 contributes to Aβ accumulation in *APOE4* carriers, accompanied by decreased ABCA1. In APOE4‐targeted replacement mice, the treatment of ABCA1 agonist, CS‐6253, activates ABCA1 and ABCA1‐mediated APOE lipidation, therefore resulting in reducing Aβ accumulation and tau hyperphosphorylation, as well as improves cognitive function [24].

### 5.9 NVU Pathologies

NVU cells including astrocytes and pericytes envelop the BMECs [259] to maintain the BBB integrity, as well as other brain cells such as microglia [187, 282] and oligodendrocytes [275]. In AD cases, these NVU cells are skewed into detrimental profile and contribute to endothelial pathologies through detrimental secretions (cytokines, chemokines, et al.). CSF level of YKL‐40, a marker of microglial inflammation is significantly higher in CSF from MCI and AD patients. The correlation between increase of YKL‐40 and BBB leakiness (indicate by increased albumin quotient) is found in AD patient [148]. In our group, we have found that canthin‐6‐one from traditional Chinese medicine Kumu can protect BEC against the damage from LPS‐induced astrocytes, including maintaining the expression of TJ, AJ, and transporter proteins as well as alleviating endothelial inflammation. The underlying mechanism involves ameliorating NFκB, STAT3, MAPK, and NLRP3 pathway [260]. Loss of pericytes and detachment from BBB are observed in AD. In APP/PS1 mice, pericyte degeneration characterized as decreased proliferation, mitochondrial damage and increased mitophagy is detected. Study also shows the involvement of activation of CD36/PINK1/Parkin pathway in pericyte degeneration [110]. Pericytes also contribute to BBB breakdown through the secretion of MMP9 [143], proangiogenic factors and proinflammatory factors [190, 194]. The critical role pericyte in BBB breakdown is especially reported in AD model carrying APOE4 allele, in which TJ proteins downregulation [155], MMP‐9 elevation [20] along with pericyte degeneration are observed [72]. MMPs also mediate the BBB disruptive effect of AD‐stimulated astrocytes and oligodendrocytes [275]. AD astrocytes or pericytes can also induce BBB disruption through the secretion of vascular endothelial growth factor (VEGF), which further triggers the downregulation of claudin‐5/occludin and leukocyte infiltration via VEGFR2 and eNOS‐mediated pathway [6, 7]. Harmful secretions from NVU cells also affect the vasoactivity of brain vessels. For example, increased secretion of prostaglandin E2 (PGE2) from astrocyte constricts capillary and arteriole respectively via modulating pericytes and vascular smooth muscle cell, which lead to decreased cerebral blood flow [45] (Figure 4). Studies have shown the therapeutic potentials of targeting NVU pathologies for BBB protection, especially astrogliosis and microgliosis. For example, L‐norvaline protects BBB integrity and alleviates cerebral amyloid angiopathy in 3 × Tg‐AD mice by alleviating astrogliosis and microgliosis [169]. Melatonin could inhibit MMP‐9 secretion from pericytes via inactivating NOTCH3/NFκB pathway, further protect BBB integrity against pericyte‐derived MMP‐9 in cerebral small vessel disease [176]. Other research also points out the therapeutic potentials of attenuating mitochondrial apoptosis in NVU cell members and carbonic anhydrases (CAs) are demonstrated potential target. Among them, pan‐CA inhibitors are approved by FDA for its effects in preventing cerebrovascular and neurovascular pathology in AD and stroke [106]. Similar findings are also observed in all‐trans retinoic acid‐treated TBI model [83]. Activated platelet‐derived growth factor CC (PDGF‐CC) contributes to BBB permeability in ischemia stroke via activating PDGFRα‐mediated signaling in astrocytes. Inhibition of PDGF‐CC/PDGFRα signaling pathway by remote ischemic conditioning restores the BBB integrity and neurological functions in thromboembolic stroke model [75] (Figure 5).

### 5.10 Other Potential Signaling

RhoA activation is a critical event during BBB hyperpermeability. Activation of RhoA‐ROCK pathway is observed in 5 × FAD mice and Aβ_1‐42_‐induced cell models. Human recombinant annexin A1 inhibits RhoA‐ROCK and reverses BBB breakdown, as well as upregulates ZO‐1 and claudin‐5 [162]. Studies also show the important role of transformer 2 alpha homolog (TRA2A)/LINC00662/ETS‐domain protein 4 (ELK4) axis in modulating BBB breakdown in AD. TRA2A is enriched in ECs in AD patients and ELK mediates inflammatory process [242]. In Aβ‐induced ECs, knockdown of TRA2A/LINC00662 induces upregulation of ELK4 and could decrease BBB permeability and upregulate TJ proteins [121]. Insulin pathway is important for cellular activities of various brain cells. Activation of insulin pathway may protect BBB integrity. It is found that high‐fat diet improves BBB integrity and cognitive function, in which upregulated insulin receptor, increased insulin signaling and lipid metabolism are involved [53]. TGR5 exhibits anti‐inflammatory, anti‐oxidative stress effects and could treat EAE [138]. BRCA1 is reported expressed in ECs and could improve endothelial survival [200]. And it is also found that deletion of BRCA1 aggravated AD pathology [213]. Upregulation of TGR5 and BRCA1 are observed in MCAO mice brain, seemingly to perform a defensive effect. In MCAO mice, treatment of TGR5 agonist activates BRCA1/Sirt1 pathway, thereafter improved BBB integrity, TJ expression and endothelial survival [113]. Hippo/YAP/TAZ pathway regulates tissue regeneration and cell proliferation [68] and PPARγ/Kruppel‐like factor 11 (KLF11) pathway, especially KLF11 could inhibit inflammatory stimuli‐induced endothelial activation [271]. These two pathways are demonstrated to modulate the expression of TJ proteins expression and protecting BBB integrity in MCAO/R mice. As we have suggested above the supportive role of Mfsd2a on BBB integrity and in AD mice, Mfsd2a is downregulated. Upregulation of Mfsd2a reverses BBB damage in subarachnoid hemorrhage rats, and such protection may attribute to the increased influx of omega‐3 fatty acids by Mfsd2a and inhibited caveolae‐based transcellular transport. PTEN/AKT/NEDD4‐2/MFSD2A axis modulates Mfsd2a level. It suggested that inhibiting NEDD4‐2‐mediated degradation of Mfsd2a by activating PTEN and AKT may be a potential therapeutic strategy to improve BBB integrity and decrease caveolae‐mediated transcytosis, which determines the restrict BBB permeability [43].

### 5.11 RNA Targeted Therapy

A series of microRNA‐targeted therapies are capable of protecting BBB integrity in ischemia stroke model or hemorrhage model by modulating the expression of BBB‐related genes. For instances, ablation of miR‐15a/16–1 could abolish its inhibition on claudin‐5 gene expression, therefore, upregulate the protein level of claudin‐5 in ischemic stroke [129]. Treatment of miR‐126‐3p inhibits the expression of VCAM‐1 gene, therefore decrease VCAM‐1‐induced vascular inflammation in cerebral hemorrhage model [62]. Brown fat enriched lncRNA 1(Blnc1) is reported upregulated in ICH brain. Blnc1 siRNA could improve BBB integrity and attenuates vascular inflammation through inhibiting PPARγ/SIRT6‐mediated FoxO3 activation in ICH brain [243]. Especially, Gao's group has developed a nano‐modulator termed siR/PIO@RP that can specifically target on damaged brain blood vessels. siR/PIO@RP is consisted of PPAR agonists and siRNA that targets RAGE expression, which has dual‐functions of activating PPARγ and downregulating RAGE. Treatment of siR/PIO@RP protects NVU system and improves cognitive functions, as well as reduces Aβ burden in APP/PS1 mice [241]. In addition, a series of chemical compounds are found capable of modulating RNA level or function. For examples, 2,4,5‐trihydroxybenzaldehyde (TDB) improves endothelial survival via suppressing miR‐34a, therefore inhibiting miR‐34a‐mediated activation of mitochondrial apoptosis in OGD‐induced cell model [114]. Moreover, ginsenoside Rg1 also exert BBB protective effects partially by inhibiting peripheral macrophage‐derived miR‐21 release, therefore suppressing brain NFκB activation in TBI mice [266].

In summary, targeting EC pathologies and BBB protection are effective in improving the cognitive functions in AD models. Attenuating inflammation or oxidative stress, autophagy, apoptosis, and pyroptosis seems effective in AD models. Especially, modulating several pathways mediated by NFκB, RhoA/ROCK, TRA2A/LINC00662, and mTOR pathway in ECs, as well as NVU pathologies especially gliosis show great therapeutic potentials. Although evidences about therapeutic effects of proangiogenic pathway, Wnt/β catenin pathway, MMPs‐mediated BBB dissociation even P‐gp or LRP‐1 mediated Aβ clearance in AD treatment are rare, their important roles in treating other neurodegenerative diseases implies great potentials in AD treatment. Compounds from natural products, approved drugs, RNA‐targeted therapies, or MSC transplantation have shown protective effects on BBB, however, it is hard to say the protective effects targeting on ECs rather than on other brain cells. BBB is a complexed structure, the cells in which mutually communicate. EC protection may results from the modulation on other brain cells. Therefore, therapies targeting on NVU pathologies such as gliosis and pericyte degeneration also improve BBB integrity. MSC transplantation can modulate NVU cells such as the expression of VEGF and MMP‐9 in astrocyte. For EC‐targeted therapies, firstly, EC‐specific targets including VCAM‐1 and Mfsd2a. C3R‐mediated VCAM‐1 and PTEN/AKT/NEDD4‐2/MFSD2A are proved as effective targets. In addition, Gao's group further suggested an EC‐targeted therapy by using nano‐system to achieve specific therapy via RAP, a peptide that can specifically bind to RAGE expressed on damaged blood vessels [241]. ABCA1 agonist, pan‐CA inhibitor, and recombinant TNF antibody can specifically modulate Aβ clearance, mitochondrial apoptosis, and vascular inflammation, implying their potentials in AD treatment. The approved drugs dabigatran, Azelnidipine, and pan‐CA inhibitor show BBB protective effects in AD and deserve further investigation to develop as auxiliary drugs for AD. In addition, more investigations are required to elucidate the contradictory findings about EC therapies, for example, which one is therapeutic, enhancement or inhibition on autophagy, and upregulation or downregulation of VEGF.

## 6 Indicators With Potential Clinical Applications

To evaluate BBB leakiness in clinic, the non‐invasive approaches are in need, such as image techniques and analysis of plasma or CSF. For image techniques, MRI image using Gd‐based contrast agent via intravenous injection is a widely used method for evaluating BBB permeability (*K*_trans_). FDG‐PET detects brain uptake of glucose and glucose metabolism, indirectly indicating the BBB functions. However, current image techniques are incapable of detecting protein distribution, such as TJ or AJ proteins, even the distribution of immune cells or plasma‐derived proteins such as fibrinogen, thrombin, and immunoglobulins in brain tissues. Plasma analysis can evaluate substance transport across BBB and vascular inflammation. CSF level of insulin reflects uptake of insulin into brain. Plasma level of n‐3 fatty acids and CSF level of DHA reflect DHA level in brain. CSF levels of fatty acids, cholesterol, and phospholipids reflect the cholesterol metabolism. Cell cholesterol efflux capacity of CSF are measured to reflect the cholesterol efflux from astrocytes. CSF levels of HNE can reflect ABCC1 ability. For vascular inflammation, the enzymatic activity of SSAO/VAP‐1 and activation of plasma protein FXII‐driven contact system can be evaluated by analyzing plasma samples. CSF levels of proinflammatory cytokines are used to reflect neuroinflammation. Especially, chemoattractants (P‐selectin, E‐selectin, ICAM‐1, VCAM‐1, PLVAP) can reflect vascular inflammation. Furthermore, several factors correlating with vascular degeneration are used to identify the progression of AD. That includes heart‐type fatty acid‐binding protein (hFABP) (associated with longitudinal atrophy of the entorhinal cortex and other LOAD‐vulnerable neuroanatomical regions), cortisol (associated with the integrity of the vascular system and risk of cardiovascular disorders), and Apolipoprotein A (Apo A) (associated with the integrity of the vascular system and risk of cardiovascular disorders) [85].

## 7 Conclusion and Perspectives

In this review, we have thoroughly described the BBB alterations including the changes in paracellular permeability, influx transporters, efflux transporters, and vascular inflammation reported in AD patients, AD transgenic animal models, and in vitro AD cell models. Preclinical and clinical evidence have both pointed out the existence of disruption of paracellular permeability, abnormality of transporter system, and vascular pathologies under AD conditions, all indicating the breakdown of BBB. Disrupted BBB is a risk factor for AD progression. As we have reviewed, increased paracellular permeability may aggravate AD pathologies by allowing the entrance of blood‐derived proinflammatory substances like fibrinogen and thrombin, which may aggravate neuroinflammation, amyloid deposition, and neurotoxicity. Dysfunctional influx transporter hampers the supply of glucose and nutrient molecules leading to hypometabolism which is harmful for neuronal activity. Downregulation or decreased activity of efflux transporters fails to eliminate neurotoxic molecules, especially Aβ species, eventually aggravating Aβ deposition and brain burdens. Therefore, repairing BBB breakdown may shed some lights on AD treatment. We summarized the therapeutic explorations in BBB protection under AD disease condition. To provide more evidences for the therapeutic potentials of BBB protection, we also included studies on other disease models, such as stroke, hemorrhage, and TBI. By inhibiting vascular inflammation, or NVU pathologies, preserving endothelial survival and BBB tightness, as well as transporter level and activity, ultimately promoted cognitive function and amyloid burden.

However, as we have noticed that the alterations of BBB are complicated and there are so many target signaling or specific targets. In addition, More evidences still need to figure out if it is an efficient therapeutic strategy for example by inhibiting VEGF/VEGFR pathway and autophagy pathway, upregulating ABCB1 (P‐gp) and ABCA1, activating Wnt/β‐catenin pathway. In addition, the onset of BBB breakdown differs in different cases. It can be detected earliest in the preclinical stage of AD or MCI patients. However, in transgenic AD models, the different onset stage of BBB breakdown is observed depending on different mutations. Therefore, treatment time needs carefully considering. Interfering BBB pathologies at early stages may exert better therapeutic effects. Taken together, BBB protection is an adjuvant therapeutic strategy, which may shed a light on the AD drug development, while more evidences are still needed to support the therapeutic potentials of BBB protection.

## Author Contributions

**Qian Yue:** conceptualization, data curation, formal analysis, investigation, resources, validation, visualization, writing – original draft. **Xinyue Leng:** validation, visualization. **Ningqing Xie:** validation, visualization. **Zaijun Zhang:** funding acquisition, writing – review and editing. **Deguang Yang:** writing – review and editing. **Maggie Pui Man Hoi:** conceptualization, funding acquisition, supervision, writing – review and editing.

## Ethics Statement

The authors have nothing to report.

## Conflicts of Interest

The authors declare no conflicts of interest.

## Data Availability

The authors have nothing to report.
